# The impact of dietary interventions on liver health biomarkers in individuals with metabolic dysfunction-associated steatotic liver disease (MASLD): a systematic literature review and meta-analysis of randomized controlled trials

**DOI:** 10.1007/s00394-025-03870-z

**Published:** 2026-02-14

**Authors:** Ute M. Stern, Gudrun Wagenpfeil, Susanne N. Weber, Saleh A. Alqahtani, Maurice Michel, Jörn M. Schattenberg

**Affiliations:** 1 Department of Internal Medicine II, Saarland University Medical Center, Saarland University, Homburg, Germany; 2 Institute of Medical Biometry, Epidemiology and Medical Informatics, Saarland University, Homburg, Germany; 3 Organ Transplant Center of Excellence, King Faisal Specialist Hospital & Research Center, Riyadh, Saudi Arabia; 4 Department of Internal Medicine II, Saarland University Medical Centre, Saarland University, 66424 Homburg, Germany

**Keywords:** Dietary interventions, Fasting, Mediterranean diet, Omega-3 fatty acids supplementation, LCHF / ketogenic diet, Metabolic dysfunction-associated steatotic liver disease (MASLD), MetALD

## Abstract

**Purpose:**

Metabolic dysfunction-associated steatotic liver disease (MASLD) and its subtype, metabolic dysfunction-associated alcohol-related liver disease (MetALD), represent the most prevalent chronic liver diseases worldwide, closely linked to unhealthy dietary patterns. Lifestyle modification is considered first-line treatment, yet the comparative effectiveness of different dietary approaches remains unclear. This systematic review and meta-analysis aimed to evaluate the impact of dietary interventions on liver health biomarkers in individuals with MASLD and MetALD.

**Methods:**

A systematic database search was conducted for randomised controlled trials (RCTs, 2018–2024). Eligible trials assessed dietary interventions in MASLD or MetALD and reported changes in alanine aminotransferase (ALT), liver stiffness, MRI-proton density fat fraction (MRI-PDFF), and controlled attenuation parameter. Data were synthesized using weighted mean differences with fixed or random effects models.

**Results:**

Sixty-eight full-text articles were included in the systematic review, of which 24 met the criteria for the meta-analysis. Since no eligible studies were identified in individuals with MetALD, the findings apply solely to people with MASLD. In studies on fasting interventions ALT (MD = − 12.47 IU/L; 95% CI − 22.03,− 2.92; *p* = 0.01; *n* = 6) and liver stiffness (MD = − 0.24 kPa; 95% CI − 0.46, − 0.03; *p* = 0.03; *n* = 4) were reduced compared to controls. The Mediterranean diet (MedDiet) resulted in significant differences in ALT (MD = − 2.93 IU/L; 95% CI − 5.68, − 0.19; *p* = 0.04; *n* = 9), liver stiffness (MD = − 0.35 kPa; 95% CI − 0.54, − 0.16; *p* = 0.00; *n* = 4), and MRI-PDFF (MD = − 1.37%; 95% CI − 2.33, − 0.40; *p* = 0.01; *n* = 5). LCHF/ketogenic diets (*n* = 6) and Omega-3 fatty acids supplementation (*n* = 4) did not significantly alter ALT.

**Conclusion:**

Fasting and MedDiet showed positive effects on surrogate biomarkers in MASLD. Larger, long-term isocaloric RCTs with standardized outcome reporting are warranted to confirm these findings.

**Supplementary Information:**

The online version contains supplementary material available at 10.1007/s00394-025-03870-z.

## Introduction

Metabolic dysfunction-associated steatotic liver disease (MASLD), formerly termed non-alcoholic fatty liver disease (NAFLD), is characterized by hepatic steatosis in conjunction with one or more cardiometabolic risk factors, such as type 2 diabetes mellitus (T2DM), obesity or dyslipidemia, in the absence of harmful levels of alcohol consumption. The disease spectrum encompasses simple steatosis, Metabolic dysfunction-associated steatohepatitis (MASH), fibrosis, cirrhosis, and MASH-related hepatocellular carcinoma (HCC) [1].

MetALD refers to a subtype of steatotic liver disease characterized by moderate alcohol intake, defined as 20–50 g/d for women and 30–60 g/d for men. Individuals with MetALD share the same metabolic risk profile as those with MASLD but consume greater amounts of alcohol without fulfilling the diagnostic criteria for alcoholic liver disease (ALD) [1]. Notably, although MASLD and MetALD share a comparable prevalence of cardiometabolic risk factors, MetALD is associated with an increased risk of all-cause mortality [1].

With a global prevalence estimated at approximately 38% [2], MASLD represents the most common chronic liver disease worldwide. It significantly contributes to the global burden of liver-disease-related mortality [3] and imposes a substantial socio-economic burden [4]. MASLD is associated with an increased risk of cardiovascular events, chronic kidney disease, hepatic and extrahepatic malignancies, and progressive liver-related complications, including end-stage liver disease and HCC. It is widely regarded as the hepatic manifestation of the metabolic syndrome, with insulin resistance being a central pathophysiological driver [5].

A Western dietary pattern has been prospectively linked with the development and progression of MASLD and is positively associated with weight gain and insulin resistance, thereby acting as a major driver of disease pathogenesis and progression [6, 7]. Given the strong association between unhealthy lifestyle behaviors and MASLD, lifestyle modification, particularly aiming at weight loss, remains the cornerstone and first-line therapeutic approach [8, 9]. Evidence suggests that a ≥ 5% reduction in body weight is necessary to reduce hepatic steatosis, 7–10% weight loss is required to attenuate hepatic inflammation, and ≥ 10% to achieve histological improvements in fibrosis [10, 11].

These therapeutic targets can be pursued through various dietary strategies, including adherence to the Mediterranean diet, time-restricted eating, low-carbohydrate high-fat diets (LCHF), or increased physical activity.

Additionally, there is growing interest in adjuvant therapies, including resveratrol, omega-3 fatty acids, herbal preparations, and agents targeting the gut microbiota, that may exert hepatoprotective effects and support dietary interventions in managing steatotic liver disease. However, a major limitation of lifestyle modifications is the difficulty of achieving and sustaining meaningful weight loss. In clinical trials involving individuals with obesity, only a minority can attain and maintain ≥ 5% weight reduction over a long time [12].

Given these challenges, this meta-analysis aims to quantitatively assess the effect size of dietary interventions on liver health biomarkers in patients with MASLD and MetALD. Specifically, it aims to determine whether certain dietary patterns confer superior benefit or whether they may be considered equally effective.

## Methods

### Search strategy

The present systematic review and meta-analysis was conducted following the Preferred Reporting Items for Systematic Reviews and Meta-Analyses (PRISMA) 2020 guidelines (Supplementary Table 3) [13].

A comprehensive systematic literature search was conducted across multiple databases in August 2024, including Medline, Embase, the International Clinical Trials Registry Platform, ClinicalTrials.gov, and Web of Science. The review focuses on randomised controlled trials (RCTs) published between 2018 and 2024 investigating any dietary intervention in MASLD treatment.

A structured search strategy was formulated using the PICO scheme, and the following Medical Subject Headings (MeSH) terms were used:

*“Non-alcoholic fatty liver disease”*,* “metabolic syndrome”*,* “alcoholic liver diseases”*,* “alcoholic fatty liver”*,* “nutrition therapy”*,* “diet therapy”*,* “Mediterranean diet”*,* “ketogenic diet”*,* “caloric restriction”*,* “fasting”*,* “intermittent fasting”*,* “dietary patterns”*,* “dietary supplements”*,* “dietary approaches to stop hypertension”*, and *“weight loss”.* The complete search strategy is presented in Supplementary Table 4.

Additionally, the reference lists of relevant review articles were screened manually to identify eligible studies. Articles identified through database search and reference screening were imported into Covidence [14], and duplicates were removed. This review was not registered in PROSPERO or any other registry.

### Eligibility criteria

Table 1 provides a summary of the inclusion and exclusion criteria applied for study selection in this systematic review and meta-analysis. The primary focus of this study was on liver health-related biomarkers, with alanine aminotransferase (ALT) levels defined as the primary outcome measure. In addition, data on secondary outcomes, including liver stiffness, magnetic resonance imaging-proton density fat fraction (MRI-PDFF), and controlled attenuation parameter (CAP), were extracted, if available, for inclusion in the meta-analysis. To be eligible for quantitative synthesis, outcomes had to be reported as mean changes (∆mean) and standard deviation (∆SD) from baseline to the end of the study.

**Table 1:** Eligibility criteria

Inclusion criteria	Exclusion criteria
Randomized controlled trials (RCTs)	Nonrandomized studies (e.g., reviews, observational studies etc.)
Male/female participants	Non-English language studies
Diagnosis of NAFLD/MASLD	Pediatric populations
Optional presence of metabolic risk factors (e.g. increased waist circumference, elevated arterial pressure, fasting glucose, serum triglycerides, cholesterol) indicative of obesity or Type 2 Diabetes Mellitus	Other causes for liver diseases – e.g., viral, autoimmune
	Restrictions regarding race or genetic background

### Study selection and data extraction

Title and abstract screening were conducted independently by two reviewers (U.M.S. and J.M.S.) using Covidence [14]. In instances where eligibility was unclear based on the abstract, studies were retained for full-text screening. Full-text screening was likewise performed independently by the same reviewers using Covidence [14]. Any discrepancies were resolved through discussion and consensus.

For each included study, the following data were systematically extracted: first author, study title, year of publication, study setting and design, eligibility criteria, sample size, participant characteristics (sex and age), details of the intervention (type and duration), and the primary outcomes reported. Extracted outcomes included liver health-related biomarkers (ALT, AST, GGT, MRI-PDFF, liver stiffness, and CAP), anthropometric indicators (body weight, BMI, waist circumference), and metabolic parameters (HbA_1c_ and HOMA-IR).

## Quality assessment

The risk of bias was assessed using the Cochrane Collaboration’s Risk of Bias Tool for Randomised Trials (ROB 2) [15]. The initial assessment was performed by one reviewer (U.M.S.), and a second reviewer (J.M.S.) independently validated the evaluations to ensure accuracy and consistency. The assessment was based on five domains: the randomisation process, deviations from the intended interventions, missing outcome data, outcome measurement, and selection of the reported result. Each domain was evaluated following the ROB 2 criteria and classified as having a low, high, or unclear risk of bias. The overall bias was determined based on the worst risk of bias in any of the domains, in accordance with the approach described by Sterne et al. [15]. The certainty of evidence for each outcome was evaluated with the Grading of Recommendations Assessment, Development, and Evaluation (GRADE) checklist using the GRADEpro tool [16].

### Statistical analysis

A quantitative meta-analysis was performed using the weighted mean difference (MD) as the effect size calculated from mean changes, standard deviation (SD), and sample sizes of the intervention and control groups. Where relevant data were missing or unclear, study authors were contacted directly to request additional information. In cases where only baseline and post-intervention means with SD were reported, the standard deviation of change (∆SD) was derived using the p-value from a paired Student’s *t*-test if available (Eq. 1). If results were presented as mean of change with 95% confidence intervals (CI), ∆SD was calculated using Eqs. 2 and 3.

1 $$\:{\sigma\:}_{diff}=\:\frac{\stackrel{-}{d}}{{t}_{1}-\:\frac{p}{2},\:df}\:\times\:\:\sqrt{n}$$

2 $$\:SE=\:\frac{upper\:limit-lower\:limit\:}{2\:\times\:t}$$

3 $$\:\varDelta\:SD=SE\:\times\:\:\sqrt{n}\:$$

Meta-analyses were performed using StatsDirect version 4.0.4, employing a fixed effects model unless significant heterogeneity was detected. The statistical heterogeneity was assessed using Cochran’s Q test and the I^2^ statistic. In the presence of substantial heterogeneity (I^2^ > 50%), a random effects model was applied.

To ensure sufficient statistical power and reliability of results, meta-analyses were conducted only for dietary interventions with data available from a minimum of four independent studies.

The presence of publication bias was assessed both statistically, using Egger’s test, and visually, through funnel plots. The results of the meta-analysis were presented as forest plots, showing the pooled weighted mean difference with corresponding 95% CI, alongside individual study estimates. The level of significance was set at *p* < 0.05. Sensitivity analyses were not conducted due to the limited number of studies per intervention.

## Results

**Fig. 1:**
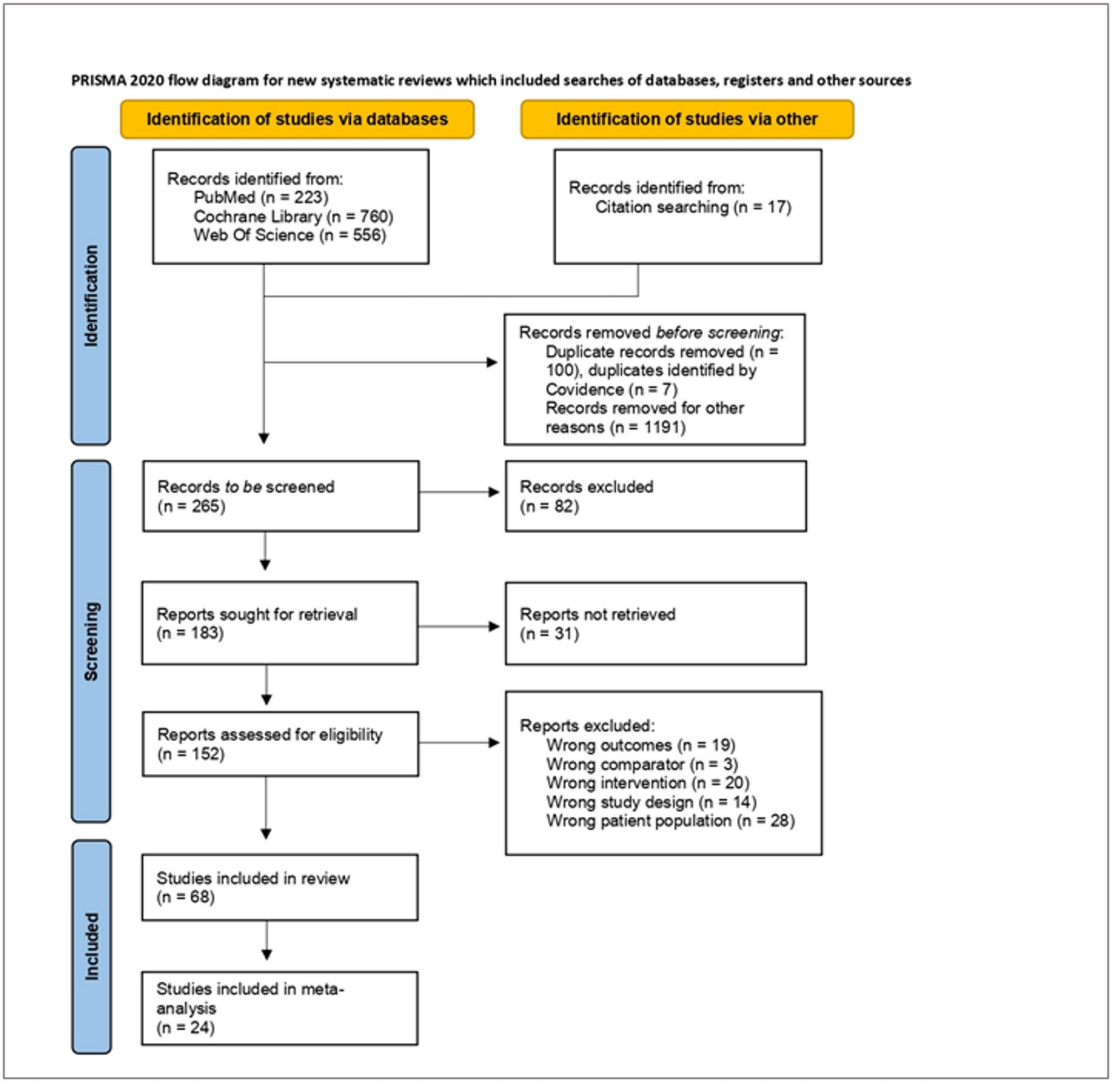
PRISMA flow diagram

**Fig. 2:**
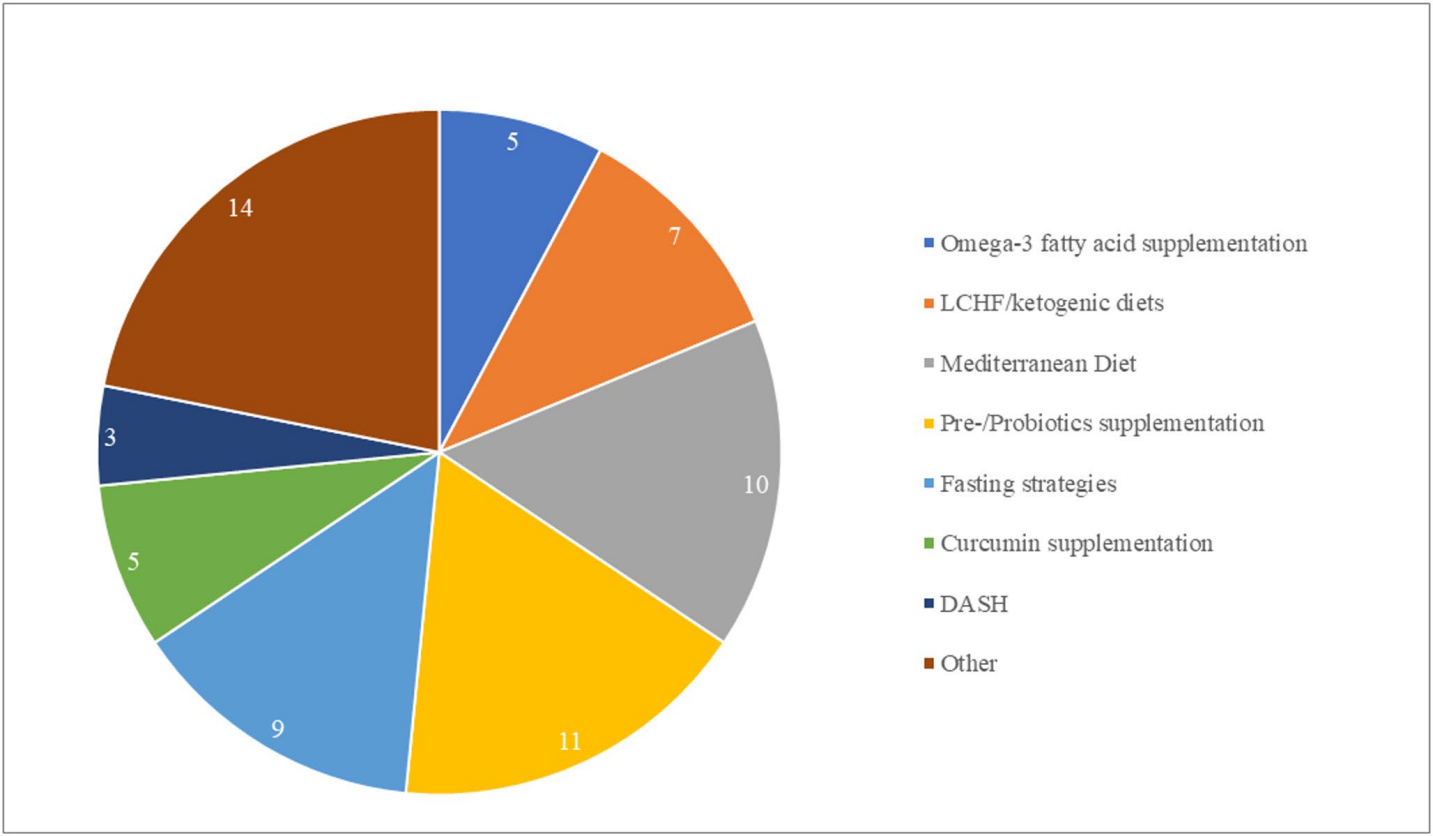
Dietary interventions included in the review (*n* = 68). *LCHF* low-carbohydrate, high-fat diet; *DASH* dietary approach to stop hypertension

The study selection process is described in the PRISMA flow diagram (Fig. 1). Out of the 1556 records initially identified, 68 full texts were included in the systematic review. Among these, 24 studies met the criteria for inclusion in the meta-analysis. A review of the literature revealed no studies investigating the use of nutritional interventions in individuals diagnosed with MetALD. The findings of this study, therefore, apply solely to people with MASLD.The identified nutritional interventions were classified into eight categories (Fig. 2): omega-3 fatty acids supplementation (*n* = 5), low-carbohydrate high-fat (LCHF)/ketogenic diets (*n* = 7), Mediterranean diet (MedDiet, *n* = 10), pre-/probiotics (*n* = 11), fasting regimens (*n* = 9), curcumin supplementation (*n* = 5), Dietary Approaches to Stop Hypertension (DASH, *n* = 3), and a heterogenous group of other interventions (*n* = 14).

The “Other” category included a range of interventions, comprising reduction of dietary gluten [17], resveratrol supplementation [18], calorie restriction [19], whole grain consumption [20], vitamin D supplementation (*n* = 2) [21, 22], nicotinamide supplementation [23], a lacto-ovo-vegetarian diet [24], cranberry supplementation [25], low free sugar diet [26] and supplementation of alpha-lipoic acid (*n* = 3, all based on the same intervention) [27–29]. Given that a minimum of four studies per intervention is required to ensure sufficient statistical power and reliability in the meta-analysis, these interventions were excluded from quantitative synthesis. We identified only three studies investigating the effects of the DASH diet on liver outcomes in individuals with MASLD. However, two studies [30, 31] are based on the same intervention. They showed that a calorie-restricted DASH diet resulted in a significantly greater reduction in ALT (MD = − 8.45 IU/L; 95% CI − 12.89, − 4.01) compared with a calorie-restricted diet only (MD = − 5.75; 95% CI − 10.46, − 1.04). Similar results were reported by Sangouni et al. [32], where the calorie-restricted DASH diet produced significantly larger reductions in ALT (MD = − 8.50 ± 8.98 IU/L vs. − 2.09 ± 7.29 IU/L; *p* = 0.002) and AST (MD = − 5.79 ± 6.83 IU/L vs. − 0.51 ± 6.62; *p* = 0.002) compared with a calorie-restricted healthy diet plan, while a non-significant trend toward lower GGT was observed.

Although eleven RCTs [33–43] evaluated the effect of pre- and probiotics supplementation on liver health biomarkers in people with MASLD, only three trials provided the data for inclusion in the meta-analysis. The effects of pre- and probiotic supplementation on liver health biomarkers in MASLD varied across the included studies. Several trials found significant reductions in liver enzymes following multi-strain probiotic interventions, containing Lactobacillus and Bifidobacterium species, and reported improved ALT, AST, and GGT [36, 38–40]. However, other studies did not observe these benefits. In these studies, the supplementation with probiotics, similarly containing Lactobacillus and Bifidobacterium species, failed to improve ALT, AST, CAP, or fibrosis score [33, 34, 42]. Prebiotics (oligofructose) were investigated in two trials, with opposing results: one observed improvements in transaminases after 16 g/day for 12 weeks [36], whereas the other reported no change in transaminases but a reduction in histologically confirmed steatosis after 8 g/day for 12 weeks followed by 16 g/day for 24 weeks [37]. Two studies evaluated synbiotic combinations. Scorletti et al. tested a fructooligosaccharide-based synbiotic for one year and found altered fecal microbiome composition without reductions in liver fat or fibrosis markers [43]. In contrast, Bakhshimoghaddam et al. reported that a synbiotic yogurt enriched with inulin for 24 weeks led to greater reductions in serum transaminases compared with conventional yogurt or control [35].

Similarly, while curcumin supplementation was assessed in five RCTs, only three met the eligibility criteria for quantitative analysis. Across the five identified trials, the study designs varied substantially, particularly regarding dosage (ranging from 50 mg/day pure curcumin [44] to 1500 mg [45]), the form of curcumin used (turmeric [46], combined with piperine [47], phospholipid-bound formulation [44] or lecithin-formulated tablets [48]), and the choice of control conditions (placebo [44, 45, 47, 48] or comparisons with chicory seed alone or the combination of turmeric and chicory seed [46]) varied greatly. The effects of curcumin supplementation on liver outcomes were inconsistent. Only Sharifi et al. [47], reported significant reductions in ALT, as well as AST, levels in the curcumin plus piperine group compared with placebo (ALT: MD = − 5.04 IU/L; 95% CI − 9.81, − 0.28 vs. MD = 6.73 IU/L; 95% CI 1.67, 11.79). In contrast, trials using higher curcumin doses alongside lifestyle modification did not show additional benefits on transaminases, CAP, or liver stiffness [45]. The study of Gharaffi et al. [46] suggested a potential synergistic effect when turmeric was combined with chicory seed, leading to reductions in ALP and GGT. However, neither phospholipid-bound nor lecithin-containing curcumin showed a superior effect on liver health biomarkers.

An overview of studies included in the meta-analysis, categorized by dietary intervention, is presented in Table 2.

**Table 2:** Characteristics of included studies

Category	Study	Intervention strategy	Characterization of liver disease	Country	*N* (% female)	Duration	Age
Fasting	Alizadeh [49]	Dinner skipping vs. no meal skipping	Vibration-Controlled Transient Elastography (VCTE)	Iran	57	3 months	43.7 (11.5)
	Ezpeleta [50]	ADF vs. control	MRI – IHTG content ≥ 5% of liver weight	USA	40 (80%)	3 months	44 (13)
	Holmer [51]	5:2-Diet vs. SoC	(1) Radiologic assessment (2) VCTE - CAP > 280 dB/m	Sweden	49 (59%)	3 months	57 (10)
	Kord-Varkaneh [54]	5:2-Diet vs. usual diet	VCTE - CAP ≥ 260 dB/m; LSM < 14 kPa	Iran	44 (39%)	3 months	46.4 (13.4)
	Lee [56]	5:2 Diet vs. SoC	(1) Biopsy – IHTG content > 5% (2) MRI-PDFF IHTG content ≥ 8%	Republic of Korea	36 (39%)	3 months	42.5 (26.9)
	Wei [57]	TRE vs. daily calorie restriction	MRI-PDFF – IHTG content ≥ 5%	China	88 (44%)	12 months	32.3 (10.5)
Low - carbohydrate, high-fat / ketogenic diet	Chen [58]	Low-carbohydrate, high fiber diet and education vs. education alone	No information	China	44 (36%)	2 months	38.1 (9.4)
	Chirapongsathorn [60]	KD vs. general lifestyle advice (DASH-diet)	Radiologic assessment or VCTE - CAP > 200 dB/m	Thailand	22 (73%)	2 months	37.4 (7.5)
	Hansen [59]	LCHF-diet vs. HCLF-diet	Biopsy	Denmark	165 (58%)	6 months	56 (10)
	Holmer [51]	LCHF vs. SoC	(1) Radiologic assessment (2) VCTE - CAP > 280 dB/m	Sweden	49 (59%)	3 months	56 (12)
	Jang [61]	LC education vs. LF education	(1) Ultrasound - abdominal ultrasound showing intrahepatic vessel blurring and increased liver parenchyma echogenicity compared with the right renal cortex in patients consuming < 140 g/week (men) or < 70 g/week (women) of alcohol (2) abnormal ALT levels > 40 IU/L	Korea	106	2 months	42.4 (13.0)
	Li [62]	KD vs. control	Elevated ALT/AST levels	China	18 (100%)	3 months	31.1 (3.6)
Mediterranean diet	Mogna-Pelaez [77]	FLiO diet vs. AHA guidelines	Ultrasound	Spain	98 (48%)	24 months	49.2 (8.9)
	Abbate [66]	MD-HMF vs. CD	Ultrasound	Spain	85 (37%)	6 months	52.9 (7.4)
	Chiurazzi [67]	MD vs. MD + nutraceuticals	No information	Italy	68 (53%)	3 months	59.8 (10.9)
	Fateh [70]	MD vs. Control	Ultrasound (≥ stage I) and elevated level of liver enzymes (not further defined)	Iraq	90 (43%)	3 months	47.3 (15.8)
	George [71]	MD vs. LFD	Ultrasound or biopsy and at least one elevated serum ALT level in the past 6 months (> 20 U/L female; > 30 U/L male)	Australia	42 (60%)	3 months	52.6 (11.7)
	Katsagoni [72]	MD vs. Control	Ultrasound, biopsy or elevated levels of ALT/GGT	Greece	42 (50%)	6 months	-
	Montemayor [73]	MD-HMF vs. CD	MRI	Spain	86 (35%)	12 months	52.3 (7.1)
	Properzi [74]	MD vs. LFD	MRS	Australia	51 (49%)	3 months	51 (13.4)
	Ristic-Medic [76]	MD vs. LFD	Ultrasound	Serbia	24	3 months	34.4 (4.7)
Omega-3 fatty acids supplementation	Climax [78]	Epeleuton (2 g/d) vs. Placebo	MRI or biopsy and ALT 1.5 to < 5 times the upper limit of normal	USA	61 (31%)	4 months	45.7 (12.0)
	Rezaei [79]	Flaxseed oil vs. sunflower oil	Ultrasound	Iran	68 (51%)	3 months	45.5 (8.7)
	Shojasaadat [80]	Fish oil (2500 mg/d) vs. control	Ultrasound	Iran	69 (45%)	3 months	41.8 (8.9)
	Yari [82]	Flaxseed vs. control	VCTE - CAP ≥ 260 dB/m	Iran	45 (47%)		45 (11)

In addition to reporting liver enzyme levels (ALT, AST, GGT), 47% of the studies included reported liver stiffness (kPa), 16% reported MRI-PDFF (%), and 26% measured CAP (dB/m) as an outcome.

### Meta-analyses for different dietary interventions

Meta-analyses were conducted to assess the effects of fasting, the LCHF/ketogenic diet, the Mediterranean diet, and omega-3 fatty acids supplementation on liver health. In the case of fasting, beyond the meta-analysis evaluating changes in ALT levels, an additional analysis was performed to investigate its impact on liver stiffness. Similarly, the Mediterranean diet was complemented by an analysis examining its influence on liver stiffness and MRI-PDFF. In contrast, for the LCHF/ketogenic diet and omega-3 fatty acids supplementation, no further meta-analyses could be conducted beyond the evaluation of their effects on ALT due to limited data availability.

### Effects of fasting interventions in people with MASLD

Among the studies included in the review, nine publications [49–57], investigated the effects of fasting interventions in people with MASLD. Of these, six studies [49–51, 54, 56, 57] were eligible for inclusion in the meta-analysis. Three studies [52, 53, 55] were excluded because they did not report the outcome as mean change $\:\pm\:$ SD, and the necessary data could not be calculated. Additionally, two publications were duplicates [52, 53].

The included studies involved a total of 314 participants, with 50% of these being female. The trials were conducted in Iran (*n* = 2), the USA, Sweden, Korea, and China, each with a duration of three months.

**Fig. 3:**
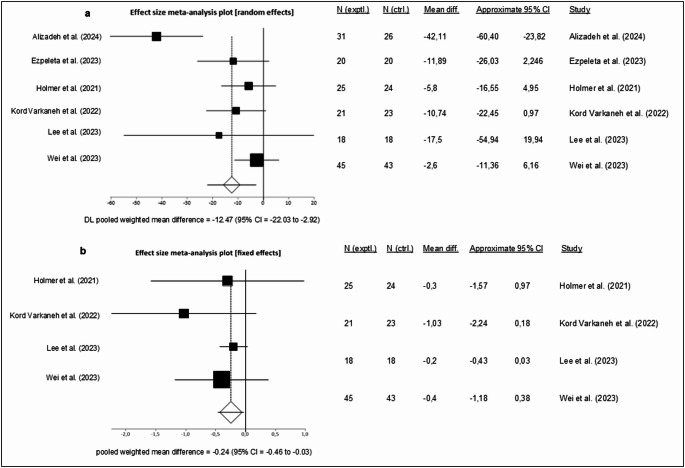
Effects of fasting interventions on liver health biomarkers in individuals with MASLD – forest plots showing the **A** change in ALT. Each square represents an individual study, with the size of the square proportional to its weight in the meta-analysis. Horizontal lines indicate the 95% confidence intervals (CI). The diamond at the bottom represents the pooled mean difference (MD) calculated using a random-effects model (MD = –12.47 IU/L; 95% CI –22.03 to –2.92) [49, 50, 51, 54, 56, 57] **B** change in liver stiffness (kPa) following fasting interventions in individuals with MASLD. Study weights are depicted by the size of the squares, and 95% CI are shown as horizontal lines. The pooled effect estimate, calculated using a fixed-effects model, is shown as a diamond (MD = –0.24 kPa; 95% CI –0.46 to –0.03) [51, 54, 56, 57]

The meta-analysis assessing the effect of fasting on ALT levels showed a statistically significant mean difference in ALT of -12.47 IU/L between the fasting intervention and control (MD: -12.47, 95% CI -22.03, -2.92, *p* = 0.01, fasting *n* = 160, control *n* = 154, Fig. 3A). There was a high level of statistical heterogeneity (I^2^ = 64%, *p* = 0.02), yet the funnel plot (Supplementary Fig. 8) did not demonstrate any publication bias (Egger’s test, *p* = 0.17).

A meta-analysis was also performed to evaluate the effect of fasting interventions on liver stiffness. Liver stiffness was analysed in four trials [51, 54, 56, 57] and the meta-analysis demonstrated a statistically significant mean difference of – 0.24 kPa (95% CI –0.46, –0.03, *p* = 0.03) between intervention and control group using a fixed effects model (fasting *n* = 109, control *n* = 108; Fig. 3B). The statistical heterogeneity of included studies was minimal (I^2^ = 0%, *p* = 0.59), and the funnel plot (Supplementary Fig. 9) did not indicate any publication bias (Egger’s test, *p* = 0.21).

### Effects of low-carbohydrate high-fat (LCHF) and ketogenic diets in people with MASLD

**Fig. 4:**
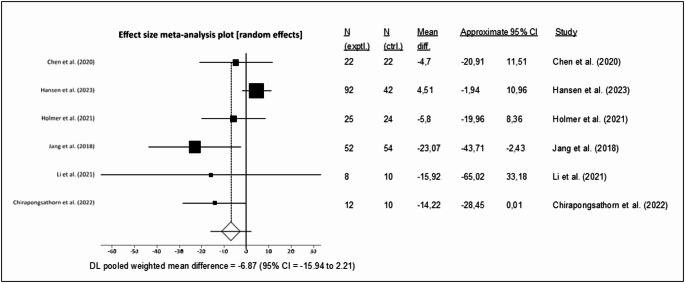
Forest plot showing the change in ALT levels in people with MASLD following a LCHF/ketogenic diet. Each study is represented by a square, the size of which reflects its weight in the meta-analysis. Horizontal lines indicate 95% CI. The diamond represents the pooled effect size using a random effects model (MD = –6.87 IU/L; 95% CI –15.93, 2.21) [est plot sh^58^–^62^]

The application of a LCHF or ketogenic diet was examined across seven clinical trials [51, 58–63], of which six [51, 58–62] were eligible for inclusion in the meta-analysis. These studies involved a total of 404 participants, 59% of whom were female. The trials were conducted in China (*n* = 2), Thailand, Denmark, Sweden, and Korea, with intervention durations ranging from two to six months. Due to data availability, a meta-analysis could only be performed on changes in ALT levels. The pooled analysis, employing a random effects model, indicated a non-significant mean difference of -6.87 IU/L between intervention and control groups (MD: -6.87 IU/L, 95% CI -15.93, 2.21, *p* = 0.14, dietary intervention *n* = 186, control *n* = 138, Fig. 4). Substantial statistical heterogeneity was observed (I^2^ = 52.3%, *p* = 0.04). The funnel plot (Supplementary Fig. 10) did not demonstrate any publication bias (Egger’s test, *p* = 0.05).

### Effects of the Mediterranean diet (MedDiet) in people with MASLD

A total of 14 studies [64–76] investigating the effect of the MedDiet in individuals with MASLD were identified. Of these, nine provided [66, 67, 70–74, 76, 77] sufficient data to be included in the meta-analysis assessing changes in ALT levels. The remaining five studies did not report their results in a format that allowed for inclusion in the meta-analysis or calculation of mean change $\:\pm\:$ SD. The studies by Marin-Alejandre et al. [64, 65], and Mogna-Peláez et al. [77] were based on the same intervention but reported outcomes at different time points. Unfortunately, only the data from Mogna-Peláez et al. [77] were available for inclusion in the meta-analysis. Similarly, the studies by Abbate et al. [66] and Montemayor et al. [73] were derived from the same intervention but differed in duration, with Abbate et al. reporting outcomes after 6 months and Montemayor et al. after 12 months, respectively.

The included studies involved a total of 586 participants, with 45% of them being female. The trials were conducted in Spain (*n* = 3), Italy, Iraq, Australia (*n* = 2), Greece, and Serbia, with a duration varying between three and 24 months.

**Fig. 5:**
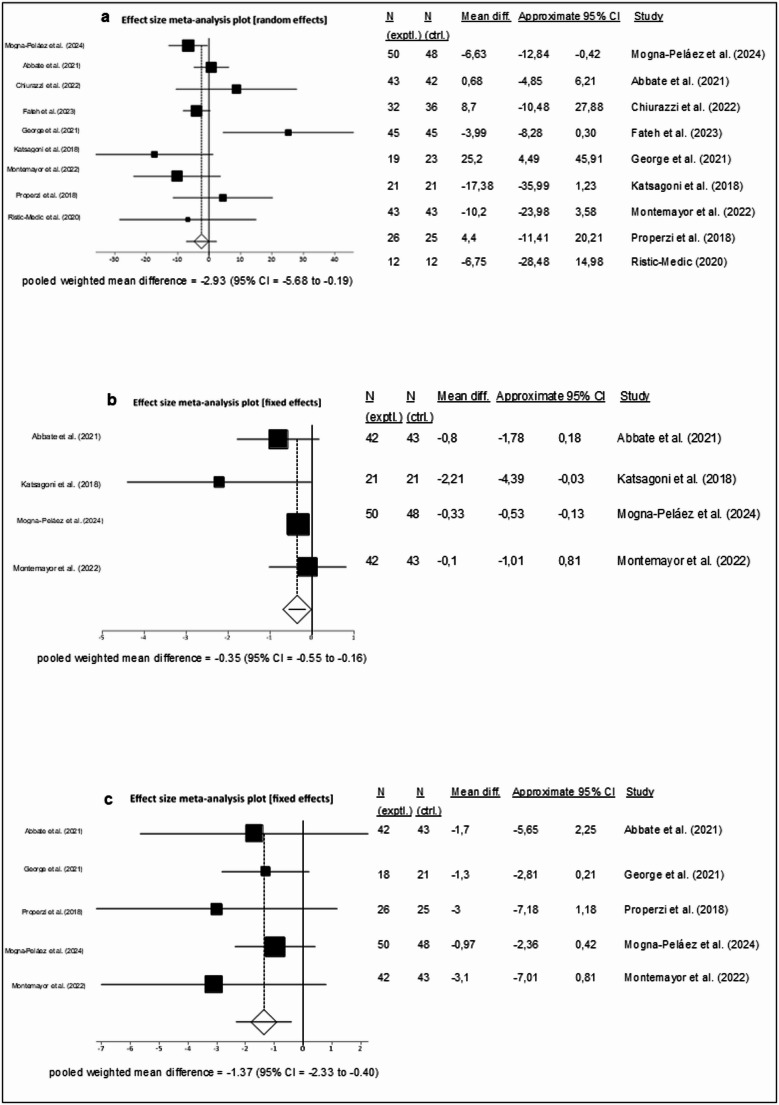
Effects of the Mediterranean diet on liver health biomarkers in individuals with MASLD – forest plots showing the **A** change in ALT levels. Each study is represented by a square, the size of which reflects its weight in the meta-analysis. Horizontal lines indicate 95% CI. The diamond represents the pooled effect size using a fixed effects model (MD = −2.93 IU/L; 95% CI −5.67, −0.19) [66, 67, 70–74, 76, 77]. **B** effect on liver stiffness. Each study is represented by a square, the size of which reflects its weight in the meta-analysis. Horizontal lines indicate 95% CI. The diamond represents the pooled effect size using a fixed effects model (MD = −0.35; 95% CI −0.54, −0.16) [66, 72, 73, 77]. **C** effect on MRI-PDFF in people with MASLD following the Mediterranean Diet. Each study is represented by a square, the size of which reflects its weight in the meta-analysis. Horizontal lines indicate 95% CI. The diamond represents the pooled effect size using a fixed effects model (MD = −1.37; 95% CI −2.33 to −0.40) [66, 71–73, 77]

The meta-analysis assessing the effect of the MedDiet on ALT levels showed a statistically significant mean difference in ALT of −2.93 IU/L between intervention and control using a fixed effects model (MD: −2.93 IU/L, 95% CI −5.68, −0.19, *p* = 0.04, MedDiet *n* = 291, control *n* = 295, Fig. 5A). There was a low level of statistical heterogeneity (I^2^ = 48.1%, *p* = 0.05), and the funnel plot (Supplementary Fig. 11) did not demonstrate a publication bias (Egger’s test, *p* = 0.57).

A meta-analysis evaluating the impact of the MedDiet on liver stiffness included four trials [66, 72, 73, 77] and demonstrated a statistically significant mean difference of -0.35 kPa (95% CI -0.54, -0.16, *p* = 0.00) in favor of the intervention, based on a fixed effects model (MedDiet *n* = 155, control *n* = 155, Fig. 5B). Statistical heterogeneity was low (I^2^ = 23.5%, *p* = 0.27), and the funnel plot (Supplementary Fig. 12) showed no evidence of publication bias (Egger’s test, *p* = 0.34).

Additionally, a meta-analysis assessing the effect of the MedDiet on MRI-PDFF was performed. This analysis included five studies [66, 71–73, 77] and showed a statistically significant mean difference of -1.37% (95% CI -2.33, -0.40, *p* = 0.01) between Mediterranean diet and control, again using a fixed effects model (MedDiet *n* = 178, control *n* = 180, Fig. 5C). Heterogeneity was negligible (I^2^ = 0%, *p* = 0.79). However, Egger’s test revealed a* p*-value of 0.04, indicating asymmetry in the funnel plot (Supplementary Fig. 13) and suggesting the potential presence of publication bias.

### Effects of an increased omega-3 fatty acids intake in people with MASLD

The impact of omega-3 fatty acids supplementation was evaluated in five [78–82] clinical trials, of which four [78–80, 82] were included in the meta-analysis. In total, these studies involved 243 participants, with 44% being female. The trials were conducted in the USA and Iran (*n* = 3) and lasted for three months.

**Fig. 6:**
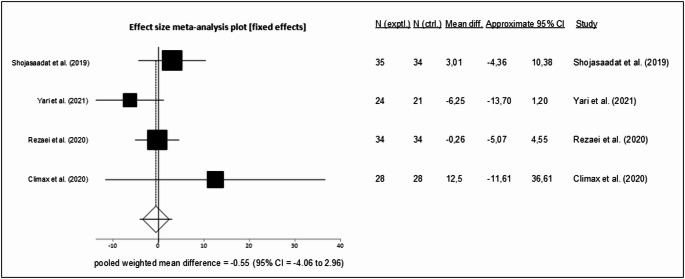
Forest plot showing the change in ALT levels in people with MASLD by supplementing omega-3 fatty acids. Each study is represented by a square, the size of which reflects its weight in the meta-analysis. Horizontal lines indicate 95% CI. The diamond represents the pooled effect size using a fixed effects model (MD = −0.55 IU/L; 95% CI −4.06, 2.96) [78–80, 82]

The meta-analysis, based on a fixed effects model, showed a non-significant mean difference of − 0.55 IU/L for change in ALT levels between the intervention and control groups (MD = − 0.55 IU/L, 95% CI − 4.06, 2.96, *p* = 0.76, intervention *n* = 121, control *n* = 117, Fig. 6). There was a low level of statistical heterogeneity (I^2^ = 29.3%, *p* = 0.24), and there was no evidence of publication bias as indicated by the funnel plot (Supplementary Fig. 14) and Egger’s test (*p* = 0.62).

### Risk of bias and GRADE assessment

**Fig. 7:**
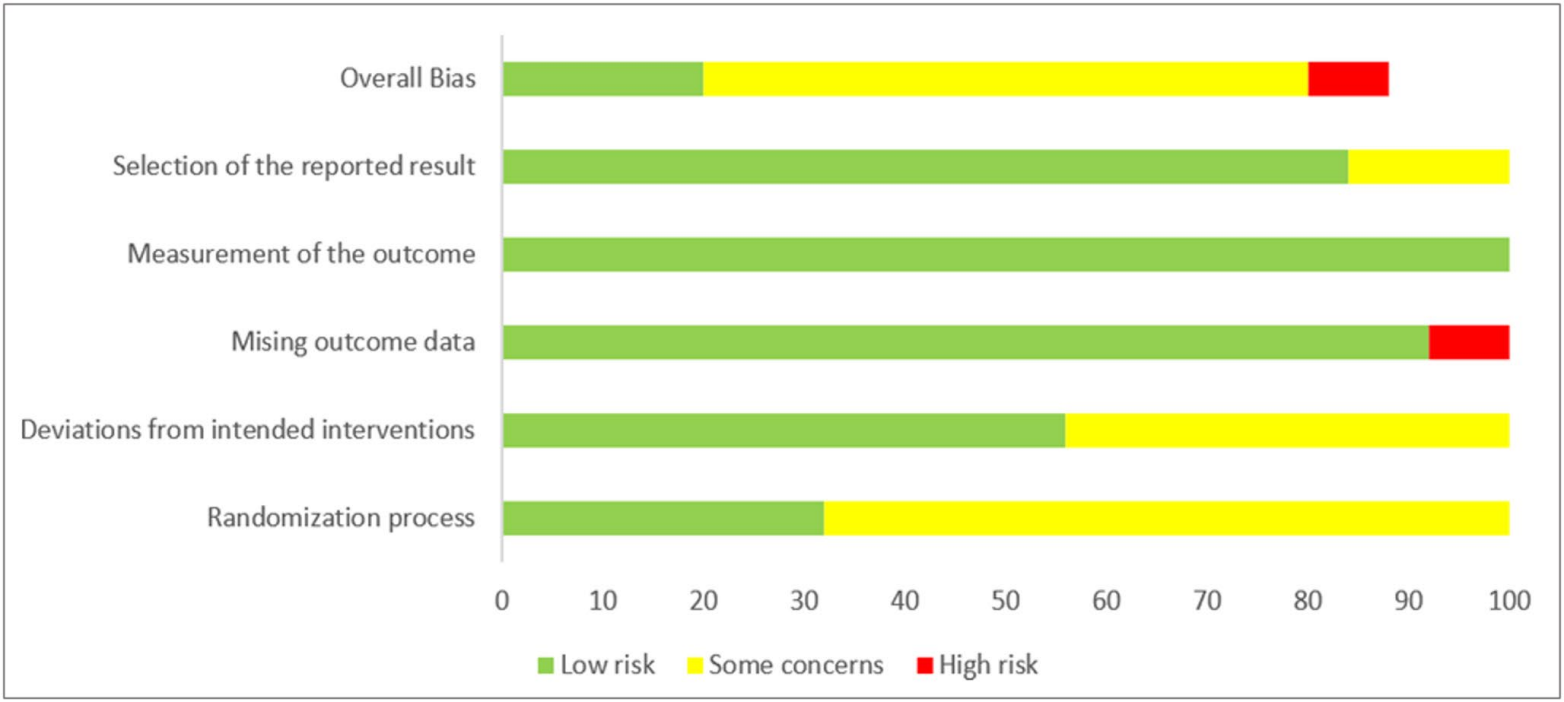
Risk of bias assessment of included RCTs across five domains and overall (green = low risk, yellow = some concerns, red = high risk)

The quality of all trials included in the meta-analyses was evaluated using the Cochrane Risk of Bias Tool (Supplementary Fig. 15), with ALT as the primary outcome. Of the included studies, seven were assessed as having a low risk of bias, fifteen were rated as raising some concerns, and two were classified as having a high risk of bias due to missing outcome data (Fig. 7).

For fasting interventions and the Mediterranean diet, the certainty of the evidence is moderate, as one study in each category was judged to be at high risk of bias. The certainty of evidence for the LCHF / ketogenic diet and the supplementation of omega-3 fatty acids was considered high (Supplementary Table 6).

## Discussion

Dietary interventions remain the cornerstone of treatment for MASLD by itself or in combination with recently approved therapies [83]. This systematic review and meta-analysis provide a comprehensive overview of current evidence on dietary interventions in individuals with metabolic dysfunction-associated steatotic liver disease. A total of 68 studies were included in the qualitative synthesis, and 24 of these provided sufficient data for meta-analysis. Eight categories of nutritional interventions were identified, but only four of them, fasting, Mediterranean diet, LCHF/ketogenic diets, and omega-3 fatty acids supplementation, had sufficient studies to allow for a quantitative synthesis, which showed that fasting interventions and the MedDiet had beneficial effects on liver health biomarkers.

Among the investigated interventions, fasting appeared to have the most robust and consistent effects. The meta-analysis demonstrated a significant effect on the reduction of ALT levels (MD = − 12.47 IU/L) and a moderate but statistically significant improvement in liver stiffness (MD = − 0.24 kPa) compared to control diet. Beyond these direct liver health biomarkers, fasting, particularly time-restricted eating, has been associated with improvements in several pathophysiological mechanisms underlying MASLD.

Meta-analyses have shown that TRE can lead to significant reductions in body weight, blood glucose, triglyceride content, and insulin resistance, which are key drivers of MASLD progression and severity [84–87]. These metabolic improvements provide a plausible mechanistic explanation for the observed hepatic benefits. Notably, the studies included in the meta-analysis were relatively homogeneous in terms of intervention duration (mostly 3 months) and design, and no evidence of publication bias was detected. However, the diversity in fasting protocols (e.g., TRE, alternate day fasting, 5:2 regimens) limits the ability to generalize the findings to a single fasting approach. Importantly, Wei et al. [57] is the only study with a duration lasting longer than three months, as such, no firm conclusions can currently be drawn regarding the long-term efficacy or sustainability of fasting interventions for MASLD, and future studies with extended follow-up are needed to assess whether benefits persist or wane over time.

The meta-analysis of LCHF and ketogenic diets demonstrated a non-significant trend toward reduced ALT levels compared to control interventions (MD = − 6.87 IU/L). There was a considerable difference in dietary interventions. The included trials applied carbohydrate targets ranging from very strict ketogenic regimens (< 10 E% [51, 60, 62] or ~ 20 E% [58, 59]) to more liberal low-carbohydrate approaches [61]. This broad range makes it difficult to ascribe potential effects to a specific dietary pattern. Importantly, the category of “low carbohydrate” does not differentiate between specific types of sugars, although fructose-containing carbohydrates (e.g., fructose, sucrose, HFCS) and glucose have been shown to exert distinct metabolic effects on hepatic physiology [88]. Fructose-rich sugars, in particular, may promote hepatic de novo lipogenesis and thereby influence liver enzymes such as ALT differently compared with glucose [89]. As the included trials did not systematically report or control for the type of carbohydrate consumed, variation in sugar composition may have contributed to inconsistent effects on ALT across studies. Future trials and meta-analyses would therefore benefit from distinguishing carbohydrate quality, not only quantity, to better clarify the role of different sugar types in modulating hepatic outcomes. Further variation between studies arose from differences in control conditions, including usual care or general lifestyle advice, and energy-restricted higher-carbohydrate diets. Only a few studies were designed as isocaloric comparisons [51, 60, 62]. Additionally, the intervention duration varied between 2 and 6 months, which again might not be sufficient to translate dietary changes into measurable improvements in liver biochemistry. Our findings are consistent with previous studies. A meta-analysis by Ahn et al. [90] comparing low-carbohydrate with low-fat diet reported no significant differences in ALT, AST, or change in hepatic fat content, concluding that both dietary strategies achieve similar improvements in MASLD. Similarly, a recent umbrella review [91] pooling three meta-analyses found no significant difference between low-carbohydrate diets and control on ALT, though a significant difference in the reduction of intrahepatic fat content based on two different meta-analyses was described. This discrepancy suggests that improvements in hepatic steatosis may not consistently be reflected in liver enzyme levels, highlighting the limitations of ALT as a sole outcome measure. Overall, the current body of evidence does not support a specific advantage of LCHF or ketogenic diets over other dietary strategies for improving ALT in MASLD.

The Mediterranean diet, as recommended by the EASL-EASD-EASO clinical practice guidelines [1], demonstrated a statistically significant, though comparatively modest, difference compared to the control in the reduction of ALT levels (MD = −2.93 IU/L). A more profound effect of the Mediterranean diet was observed by Haigh et al. with a mean difference of 6.54 IU/L [92]. In congruence with our findings on the reduction of liver stiffness (MD = − 0.35 kPa), both Del Bo et al. [93] (MD = −0.42 kPa) and Haigh et al. [92] (MD = −0.75 kPa) reported a significant difference between the groups. Beyond hepatic biomarkers, a beneficial effect on insulin resistance, as measured by HOMA-IR, was noted. However, no significant changes in BMI or waist circumference, both considered key therapeutic targets, were detected [94]. These findings suggest that the Mediterranean diet may improve hepatic steatosis independently of body weight or visceral fat reduction, and instead, the beneficial effect is attributable to its high content of functional nutrients such as virgin olive oil, polyunsaturated fatty acids, and polyphenols [95].

The included studies varied in study design and scope, ranging from small pilot RCTs [67, 74] to larger multicenter interventions [73, 77]. Control conditions were heterogeneous, encompassing low-fat, calorie-restricted regimens, standard of care, and general healthy eating advice. Furthermore, in some trials, the intervention was embedded in broader lifestyle programs that included physical counselling [66, 73]. Despite these differences, the consistency of effects across different outcome measures and the low statistical heterogeneity support the reliability of these findings. Nevertheless, only a subset of studies employed isocaloric control conditions, raising the possibility that observed benefits partly reflect differences in energy deficit rather than diet composition alone. The observed difference in MRI-PDFF (MD − 1.34%) between intervention and control group supports a beneficial effect of the Mediterranean diet; however, this result should be interpreted with caution, given the possible publication bias as indicated by the Egger’s test (*p* = 0.041). Its significance is limited due to the small number of studies included in the analysis; however, the possibility of publication bias cannot be excluded. Thus, while the overall evidence supports the recommendation of the Mediterranean diet in MASLD management, further large-scale, long-term, isocaloric RCTs with standardized protocols are needed to distinguish the specific effects of diet composition from those of caloric restriction and weight loss.

The hypothesis that the beneficial effects of the Mediterranean diet are primarily attributable to its functional components cannot be fully substantiated by this meta-analysis [95]. In particular, supplementation with omega-3 fatty acids alone did not result in a significant difference compared to placebo on ALT levels (MD = − 0.55 IU/L), and the available data do not support a clinically relevant effect on liver enzymes. The failure of omega-3 fatty acids to decrease ALT levels in MASLD patients may be due to short observation times in the included studies. This aligns with findings from other meta-analyses. For example, Lu et al. [96] observed only a trend towards improvement in ALT and AST with omega-3 fatty acids supplementation, while a significant reduction compared to the control group was reported exclusively for GGT. Similarly, Parker et al. [97] found no significant effect on ALT. He et al. [98] reported a beneficial effect when supplementing with ≥ 3 g of omega-3 fatty acids, though this result was based on only three studies, whereas doses < 3 g showed no effect on ALT compared to control. Finally, Yan et al. [99] demonstrated significant improvements in ALT, AST, and GGT based on data from up to 14 different trials; however, their analysis also included pediatric populations, which distinguishes it from the present study. Although preclinical studies have shown promising results, the small number of clinical trials, combined with variations in dosage, formulations, and background diets, limits the ability to draw definitive conclusions regarding clinical efficacy.

A major limitation of this study is the substantial variability in study design, intervention strategies, diagnosis of liver disease, and reporting of outcomes of included studies. Most trials included had small sample sizes, short durations, and variability in intervention protocols, which limits the generalizability of the findings. Moreover, the predominance of short-term interventions (typically ≤ 6 months) and the absence of long-term follow-up data constrain conclusions about sustained benefits for liver health. Although many studies examined changes in ALT, which is a useful but indirect marker, fewer reported imaging-based outcomes such as liver stiffness or MRI-PDFF, which more directly reflect hepatic fat content and fibrosis, respectively. Furthermore, only a quarter of the studies reported the CAP-value. The use of ALT as a marker for assessing hepatic health is questionable, as it is not a reliable predictor of liver disease [100]. In order to enhance the reliability of the results, it is essential to ensure consistent outcome reporting of MRI and VCTE measurements. A key limitation of the available evidence is the focus on surrogate biomarkers rather than clinical outcomes. Owing to substantial heterogeneity in patient-reported outcome measures and the limited availability of histological data, pooled analyses beyond these surrogates were not feasible. While this is a limitation, the use of surrogate endpoints is common in slowly progressive diseases such as MASLD. Notably, the Look AHEAD trial [101] showed that even intensive lifestyle modification did not reduce cardiovascular events in individuals with type 2 diabetes, despite significant weight loss and metabolic improvements. This highlights the fact that short- to mid-term biomarker changes do not necessarily translate into hard clinical outcomes. Nevertheless, surrogate markers can provide valuable information. For instance, the REGENERATE trial [102] found that a 17 IU/L decrease in ALT predicted histological improvement after one year of obeticholic acid treatment. The dietary interventions assessed here were shorter and produced smaller effects, with fasting interventions lowering ALT by approximately 12.5 IU/L. Such modest short-term changes are unlikely to have immediate clinical relevance, especially given natural fluctuations in ALT. Still, sustainable dietary changes may provide long-term benefits by improving metabolic health and slowing liver disease progression. Although the observed effects are small, their potential clinical value lies in the cumulative impact of lifestyle modification when maintained over time The exclusion of interventions with fewer than four trials, while methodologically justified for meta-analytical robustness, may have omitted potentially promising approaches (e.g., curcumin, probiotics, DASH diet). The robustness of conclusions about publication bias is limited, since Egger’s test lacks power when only a small number of studies are available. Although we additionally performed a visual inspection of funnel plots, the possibility of publication bias cannot be fully ruled out particularly for the meta-analyses on the effects of fasting interventions and the Mediterranean diet on liver stiffness, as well as on the impact of omega-3 fatty acid supplementation on ALT levels. These findings are based on a very limited evidence, with only four studies contributing to the analysis. Notably, no studies were identified that targeted individuals with metabolic dysfunction-associated alcohol-related liver disease (MetALD), highlighting a clear research gap.

The overall quality of included studies varied. Although most trials were assessed as having low or moderate risk of bias, two studies were rated as high risk due to missing outcome data.

In conclusion, future RCTs with longer follow-up and standardised outcome reporting are needed to confirm and expand on these findings. In clinical practice, fasting and the Mediterranean diet may be considered valid options for MASLD management.

## Supplementary Information

Below is the link to the electronic supplementary material.

Supplementary Material 1

## Data Availability

All data extracted from the included studies and the analytic code used for meta-analyses are available from the corresponding author upon reasonable request.

## Abbreviations

AHA: Amercian heart association
ADF: alternate day fasting
ALT: alanine aminotransferase
AST: aspartate aminotransferase
CD: conventional diet
FLiO: Fatty liver in obesity (Mediterranean-based diet)
GGT: gamma-glutamyltransferase
HCLF: high-carb low-fat diet
ICR: intermittent calorie restriction
KD: ketogenic diet
LCD: low-carbohydrate diet
LCHF: low-carbohydrate high-fat diet
LFD: low-fat diet
MedDiet: Mediterranean Diet
MedDiet-HMF: Mediterranean Diet-high meal frequency
MRI-PDFF: magnetic resonance imaging-proton density fat fraction
SOC: standard of care
TRE: time-restricted eating
