# Supplementary material for: The impact of dietary interventions on liver health biomarkers in individuals with metabolic dysfunction-associated steatotic liver disease (MASLD): a systematic literature review and meta-analysis of randomized controlled trials
Source: Eur J Nutr. 2026 Feb 14;65(2):58. doi: 10.1007/s00394-025-03870-z (PMC12906548; doi:10.1007/s00394-025-03870-z)
Supplement: Supplementary file 1 — Supplementary Material 1 [file 394_2025_3870_MOESM1_ESM.docx]

**Supplementary materials**

Contents

[Table 3 Preferred Reporting Items for Systematic Reviews and Meta-Analyses (PRISMA) checklist 2](#_Toc208932103)

[Table 4 Search queries for PubMed, Cochrane Library, and Web of Science 4](#_Toc208932104)

[Table 5 Table of excluded studies with rationale 5](#_Toc208932105)

[References of excluded studies 5](#_Toc208932106)

[Fig. 8 Funnel Plot assessing potential publication bias regarding the effect of fasting interventions on serum ALT in individuals with MASLD. The vertical line represents the pooled effect size, and the diagonal lines indicate the 95% confidence limits 11](#_Toc208932107)

[Fig. 9 Funnel plot assessing potential publication bias regarding the effect of fasting interventions on liver stiffness in individuals with MASLD. The vertical line represents the pooled effect size, and the diagonal lines indicate the 95% confidence limits. 12](#_Toc208932108)

[Fig. 10 Funnel Plot assessing potential publication bias regarding the effect of a LCHF/ketogenic diet on serum ALT in individuals with MASLD. The vertical line represents the pooled effect size, and the diagonal lines indicate the 95% confidence limits. 13](#_Toc208932109)

[Fig. 11 Funnel Plot assessing potential publication bias regarding the effect of the Mediterranean diet on serum ALT levels in individuals with MASLD. The vertical line represents the pooled effect size, and the diagonal lines indicate the 95% confidence limits. 14](#_Toc208932110)

[Fig. 12 Funnel Plot assessing potential publication bias regarding the effect of the Mediterranean diet on liver stiffness in individuals with MASLD. The vertical line represents the pooled effect size, and the diagonal lines indicate the 95% confidence limits. 15](#_Toc208932111)

[Fig. 13 Funnel Plot assessing potential publication bias regarding the effect of the Mediterranean diet on MRI-PDFF in individuals with MASLD. The vertical line represents the pooled effect size, and the diagonal lines indicate the 95% confidence limits. 16](#_Toc208932112)

[Fig. 14 Funnel Plot assessing potential publication bias regarding the effect of the supplementation of omega-3 fatty acids on serum ALT levels in individuals with MASLD. The vertical line represents the pooled effect size, and the diagonal lines indicate the 95% confidence limits. 17](#_Toc208932113)

[Fig. 15 Quality assessment of the included randomized controlled trials. 18](#_Toc208932114)

[Table 6 GRADE assessment 18](#_Toc208932115)

# Table 3 Preferred Reporting Items for Systematic Reviews and Meta-Analyses (PRISMA) checklist

| **Section and Topic** | **Item #** | **Checklist item** | **Location where item is reported** |
| --- | --- | --- | --- |
| **TITLE** | | |  |
| Title | 1 | Identify the report as a systematic review. | p. 1 |
| **ABSTRACT** | | |  |
| Abstract | 2 | See the PRISMA 2020 for Abstracts checklist. | p.3 |
| **INTRODUCTION** | | |  |
| Rationale | 3 | Describe the rationale for the review in the context of existing knowledge. | p. 5+6 |
| Objectives | 4 | Provide an explicit statement of the objective(s) or question(s) the review addresses. | p. 6 |
| **METHODS** | | |  |
| Eligibility criteria | 5 | Specify the inclusion and exclusion criteria for the review and how studies were grouped for the syntheses. | p. 8 + Table 1 |
| Information sources | 6 | Specify all databases, registers, websites, organisations, reference lists and other sources searched or consulted to identify studies. Specify the date when each source was last searched or consulted. | p. 7 |
| Search strategy | 7 | Present the full search strategies for all databases, registers and websites, including any filters and limits used. | p. 7 + Table S2 |
| Selection process | 8 | Specify the methods used to decide whether a study met the inclusion criteria of the review, including how many reviewers screened each record and each report retrieved, whether they worked independently, and if applicable, details of automation tools used in the process. | p. 7+8 |
| Data collection process | 9 | Specify the methods used to collect data from reports, including how many reviewers collected data from each report, whether they worked independently, any processes for obtaining or confirming data from study investigators, and if applicable, details of automation tools used in the process. | p. 7+8 |
| Data items | 10a | List and define all outcomes for which data were sought. Specify whether all results that were compatible with each outcome domain in each study were sought (e.g. for all measures, time points, analyses), and if not, the methods used to decide which results to collect. | p. 8 |
|  | 10b | List and define all other variables for which data were sought (e.g. participant and intervention characteristics, funding sources). Describe any assumptions made about any missing or unclear information. | p. 8 |
| Study risk of bias assessment | 11 | Specify the methods used to assess risk of bias in the included studies, including details of the tool(s) used, how many reviewers assessed each study and whether they worked independently, and if applicable, details of automation tools used in the process. | p. 9 |
| Effect measures | 12 | Specify for each outcome the effect measure(s) (e.g. risk ratio, mean difference) used in the synthesis or presentation of results. | p. 9+10 |
| Synthesis methods | 13a | Describe the processes used to decide which studies were eligible for each synthesis (e.g. tabulating the study intervention characteristics and comparing against the planned groups for each synthesis (item #5)). | p. 10 |
|  | 13b | Describe any methods required to prepare the data for presentation or synthesis, such as handling of missing summary statistics, or data conversions. | p. 10 |
|  | 13c | Describe any methods used to tabulate or visually display results of individual studies and syntheses. | p. 10 |
|  | 13d | Describe any methods used to synthesize results and provide a rationale for the choice(s). If meta-analysis was performed, describe the model(s), method(s) to identify the presence and extent of statistical heterogeneity, and software package(s) used. | p. 10 |
|  | 13e | Describe any methods used to explore possible causes of heterogeneity among study results (e.g. subgroup analysis, meta-regression). | p. 9+10 |
|  | 13f | Describe any sensitivity analyses conducted to assess robustness of the synthesized results. | p. 10 |
| Reporting bias assessment | 14 | Describe any methods used to assess risk of bias due to missing results in a synthesis (arising from reporting biases). | p. 9 |
| Certainty assessment | 15 | Describe any methods used to assess certainty (or confidence) in the body of evidence for an outcome. | p. 9 |
| **RESULTS** | | |  |
| Study selection | 16a | Describe the results of the search and selection process, from the number of records identified in the search to the number of studies included in the review, ideally using a flow diagram. | p. 11 |
|  | 16b | Cite studies that might appear to meet the inclusion criteria, but which were excluded, and explain why they were excluded. | Table S3 |
| Study characteristics | 17 | Cite each included study and present its characteristics. | Table 2 |
| Risk of bias in studies | 18 | Present assessments of risk of bias for each included study. | Figure S8 |
| Results of individual studies | 19 | For all outcomes, present, for each study: (a) summary statistics for each group (where appropriate) and (b) an effect estimate and its precision (e.g. confidence/credible interval), ideally using structured tables or plots. | p. 12 ff |
| Results of syntheses | 20a | For each synthesis, briefly summarise the characteristics and risk of bias among contributing studies. | p. 12-16 |
|  | 20b | Present results of all statistical syntheses conducted. If meta-analysis was done, present for each the summary estimate and its precision (e.g. confidence/credible interval) and measures of statistical heterogeneity. If comparing groups, describe the direction of the effect. | p. 112-16 |
|  | 20c | Present results of all investigations of possible causes of heterogeneity among study results. | p. 12-16 |
|  | 20d | Present results of all sensitivity analyses conducted to assess the robustness of the synthesized results. | NA |
| Reporting biases | 21 | Present assessments of risk of bias due to missing results (arising from reporting biases) for each synthesis assessed. | Figure 7 + S8 |
| Certainty of evidence | 22 | Present assessments of certainty (or confidence) in the body of evidence for each outcome assessed. | Table S4 |
| **DISCUSSION** | | |  |
| Discussion | 23a | Provide a general interpretation of the results in the context of other evidence. | p. 16-21 |
|  | 23b | Discuss any limitations of the evidence included in the review. | p. 17-21 |
|  | 23c | Discuss any limitations of the review processes used. | p. 21 |
|  | 23d | Discuss implications of the results for practice, policy, and future research. | p. 21 |
| **OTHER INFORMATION** | | |  |
| Registration and protocol | 24a | Provide registration information for the review, including register name and registration number, or state that the review was not registered. | p. 7 |
|  | 24b | Indicate where the review protocol can be accessed, or state that a protocol was not prepared. | NA |
|  | 24c | Describe and explain any amendments to information provided at registration or in the protocol. | NA |
| Support | 25 | Describe sources of financial or non-financial support for the review, and the role of the funders or sponsors in the review. | p. 23 |
| Competing interests | 26 | Declare any competing interests of review authors. | p. 1 |
| Availability of data, code and other materials | 27 | Report which of the following are publicly available and where they can be found: template data collection forms; data extracted from included studies; data used for all analyses; analytic code; any other materials used in the review. | p. 23 |

# Table 4 Search queries for PubMed, Cochrane Library, and Web of Science

| Medline – via PubMed |
| --- |
| „MASLD patients“ [TIAB] OR MASLD [TIAB] OR „metabolic dysfunction associated fatty liver disease“ [TIAB] OR „MetALD“ [TIAB] OR „NAFLD patients“ [TIAB] OR NAFLD [TIAB] OR „Metabolic Syndrome“ [TIAB] OR „Fatty Liver Disease“ [TIAB] OR „Fatty Liver“ [TIAB] OR „steatotic liver disease“ [TIAB] OR „alcoholic liver disease“ [TIAB] OR „non-alcoholic steatohepatitis“ [TIAB] OR NASH [TIAB] „non-alcoholic fatty liver disease“ [MH] OR „Fatty Liver“ [MH] OR „Liver diseases, Alcoholic“ [MH] OR „Fatty Liver, Alcoholic“ [MH] OR „Metabolic Syndrome“ [MH] AND „Liver“ [MH]    „Dietary intervention" [TIAB] OR „dietary modification" [TIAB] OR „nutritional intervention" [TIAB] OR „dietary treatment" [TIAB] OR „Nutritional therapy" [TIAB] OR „caloric restriction" [TIAB] OR „Time restricted eating" [TIAB] OR „Mediterranean diet" [TIAB] OR „Low carb high fat diet" [TIAB] OR „low fat diet" [TIAB] OR „ketogenic diet" [TIAB] OR „very low carb ketogenic diet" [TIAB] OR „dietary approaches to stop hypertension"[TIAB] OR DASH [TIAB] OR oat [TIAB] OR Supplementation [TIAB] OR „Dietary Strategies“ [TIAB] OR „Nutrition therapy" [MH] OR „Diet therapy" [MH] OR „Diet, Mediterranean" [MH] OR „Diet, Ketogenic" OR „caloric restriction" [MH] OR Fasting [MH] OR „Dietary Supplements" [MH] OR „Dietary patterns" OR „intermittent fasting" [MH]    „nutritional counselling“ [TIAB] OR „healthy diet“ [TIAB] OR control [TIAB]    „weight loss“ [TIAB] OR „Losing weight“ [TIAB] OR „Fat reduction“ [TIAB] OR „Body fat reduction“ [TIAB] OR „Liver Fat reduction“ [TIAB] OR „liver stiffness“ [TIAB] OR „hepatic fat content“ [TIAB] OR „serum liver enzymes“ [TIAB] OR „insulin sensitivity“[TIAB] OR „serum triglycerides“ [TIAB] OR „weight loss“ [MH] |
| Embase, the International Clinical Trials Registry Platform, ClinicalTrials.gov – via Cochrane Library |
| "Non-alcoholic Fatty liver disease" OR "Fatty Liver" OR "alcoholic liver disease" OR "metabolic-dysfunction associated steatotic liver disease" OR "NAFLD" OR "MASLD" OR "steatotic liver disease"    "Nutrition Therapy" OR "Diet Therapy" OR "Mediterranean Diet" OR "ketogenic Diet" OR Fasting OR "Dietary Strategies" OR "caloric restriction" OR "Dietary Intervention" OR "Dietary modification" OR "Time restricted eating" OR "Low Carb High fat diet" OR "intermittent fasting" OR "Dietary Patterns" OR Supplementation    „nutritional counselling“ OR „healthy diet“ OR control    "Liver fat reduction" OR "hepatic fat content" OR "serum liver enzymes" OR "liver stiffness" OR "serum triglycerides" OR "weight loss“ |
| Web of Science |
| "Non-alcoholic Fatty liver disease" OR "Fatty Liver" OR "alcoholic liver disease" OR "metabolic-dysfunction associated steatotic liver disease" OR "NAFLD" OR "MASLD" OR "steatotic liver disease"    "Nutrition Therapy" OR "Diet Therapy" OR "Mediterranean Diet" OR "ketogenic Diet" OR Fasting OR "Dietary Strategies" OR "caloric restriction" OR "Dietary Intervention" OR "Dietary modification" OR "Time restricted eating" OR "Low Carb High fat diet" OR "intermittent fasting" OR "Dietary Patterns" OR Supplementation    „nutritional counselling“ OR „healthy diet“ OR control    "Liver fat reduction" OR "hepatic fat content" OR "serum liver enzymes" OR "liver stiffness" OR "serum triglycerides" OR "weight loss“ |

# Table 5 Table of excluded studies with rationale

| Reason for exclusion (number) | Reference of excluded studies |
| --- | --- |
| no full text (n = 31) | [1-31] |
| Wrong comparator (n = 3) | [32-34] |
| Wrong study design (n = 14) | [35-48] |
| Wrong outcomes (n = 19) | [49-67] |
| Wrong intervention (n = 20) | [68-86] |
| Wrong patient population (n = 28) | [87-113] |

# References of excluded studies

1. Aggarwal, D., et al., *Effectiveness of Time-restricted Intermittent Fasting in Patients with Non-alcoholic Fatty Liver Disease - A Randomized Controlled Trial.* Journal of clinical and experimental hepatology, 2023. **13**: p. S137.

2. Aggarwal, D., et al., *Calorie restriction by time restricted intermittent fasting is better than standard calorie restriction in improving the metabolic profile and hepatic fibrosis in patients with non-alcoholic fatty liver disease.* Journal of hepatology, 2023. **78**: p. S55.

3. Asghari, S., et al., *Comparison of Calorie-Restricted Diet and Resveratrol Supplementation on Anthropometric Indices, Metabolic Parameters, and Serum Sirtuin-1 Levels in Patients With Nonalcoholic Fatty Liver Disease: A Randomized Controlled Clinical Trial.* Journal of the American College of Nutrition, 2018. **37**(3): p. 223-233.

4. Bozzetto, L., et al., *De novo lipogenesis mediates beneficial effects of isoenergetic dietary interventions on fatty liver: insights from the MEDEA randomized clinical trial.* Atherosclerosis, 2022. **355**: p. 23‐24.

5. Cano Contreras, A.D., et al., *Efficacy of the regional Mexican diet versus the Mediterranean diet in patients with MASLD: a 24-week non-inferiority trial.* Revista espanola de enfermedades digestivas, 2024.

6. Chayanupatkul, M., et al., *The Efficacy of Oligonol in Nonalcoholic Fatty Liver Disease: A Randomized Double-Blinded Placebo-Controlled Trial.* Journal of Integrative and Complementary Medicine, 2022. **28**(11): p. 904-908.

7. Colletta, C., A. Colletta, and G. Placentino, *Lifestyle and silymarin: a fight against liver damage in NAFLD associated - prediabetic disease.* Journal of diabetes and metabolic disorders, 2020. **19**(2): p. 883‐894.

8. Contreras, A.D.C., et al., *REGIONAL MEXICAN DIET OR MEDITERRANEAN DIET FOR PATIENTS WITH MAFLD, WHICH ONE IS BETTER? RESULTS AT 12 AND FOR 24 WEEKS.* Gastroenterology, 2023. **164**(6): p. S‐1309.

9. Della Pepa, G., et al., *Effect of a portfolio diet targeting multiple dietary components on liver fat content in individuals with type 2 diabetes: an 8-week randomised controlled clinical trial.* Diabetologia, 2019. **62**: p. S92.

10. Della Pepa, G., et al., *Treating Non-Alcoholic Fatty Liver Disease In Patients With Type 2 Diabetes By Targeting Multiple Dietary Components: the Portfolio Diet.* Atherosclerosis, 2019. **287**: p. e117.

11. Devarajan, A., et al., *Effect of Tocotrienol on Liver Enzymes, Fatty Liver and Liver Stiffness in People with Type 2 Diabetes and NAFLD : a Pilot Study Based on Biochemical and Transient Elastography Parameters.* Journal of the Indian Medical Association, 2023. **121**(6): p. 14‐18.

12. Durrer, C., et al., *The effect of a 16-week diet intervention with or without differing amounts of exercise volume on hepatic fat content in people with type 2 diabetes.* Diabetologia, 2022. **65**: p. S62.

13. Ezpeleta, M., et al., *Effect of Diet Combined With Exercise on Body Weight in Adults With Obesity and NAFLD.* Obesity (Silver Spring, Md.), 2020. **28**(SUPPL 2): p. 85.

14. Ezpeleta, M., et al., *Alternate Day Fasting Combined With Endurance Exercise for the Treatment of Fatty Liver Disease.* Obesity (Silver Spring, Md.), 2022. **30**: p. 286.

15. George, E.S., et al., *The effect of a Mediterranean diet and low-fat diet on intrahepatic fat, liver stiffness and insulin resistance in patients with non-alcoholic fatty liver disease: preliminary findings from the MEDINA Trial.* Journal of gastroenterology and hepatology, 2018. **33**: p. 141‐142.

16. Grove, J., et al., *Effect of low glycemic index carbohydrate consumption in non-alcoholic fatty liver disease: a community-based pilot randomised controlled trial and mechanistic study.* Hepatology (Baltimore, Md.), 2020. **72**(1 SUPPL): p. 369A.

17. Haigh, L., et al., *A meditarranean diet intervention has beneficial effects on biomarkers of cardiovascular risk and hepatic fibrosis in non-alcoholic fatty liver disease (NAFLD).* Journal of hepatology, 2022. **77**: p. S733‐S734.

18. Hamza, M., et al., *Epeleuton, a novel synthetic second-generation n-3 fatty acid, decreased triglycerides, improved glycémie control and decreased markers of inflammation in a phase 2 exploratory study.* Circulation, 2019. **140**.

19. Hassanian-Fard, S., K. Jalali-Dehkordi, and H. Rahimi, *The Effect of Combined Training with Dietary Approaches to Stop Hypertension (DASH) on Liver Damage Indices in Patients with Non-Alcoholic Fatty Liver Disease.* Journal of isfahan medical school, 2021. **39**(634): p. 533‐541.

20. Hellmann, P., et al., *No effect of curcumin on liver fat content in obese individuals.* Hepatology (Baltimore, Md.), 2021. **74**(SUPPL 1): p. 1118A‐1119A.

21. Karimi, M., et al., *The effects of intermittent fasting diet in comparison with low-calorie diet on lipid profile, glycemic status, and liver fibrosis in patients with non-alcoholic fatty liver (NAFLD): a study protocol for a randomized controlled clinical trial.* BMC nutrition, 2023. **9**(1): p. 145.

22. Kim, H.Y., et al., *EFFECT OF 12-WEEK INTERMITTENT CALORIE RESTRICTION ON LIVER FAT CONTENT IN COMPARISON WITH STANDARD-OF-CARE IN PATIENTS WITH NAFLD.* Hepatology (Baltimore, Md.), 2023. **78**: p. S1234.

23. Kobyliak, N., et al., *Beneficial effects of probiotic combination with omega-3 fatty acids in NAFLD: a randomized clinical study.* Minerva Medica, 2018. **109**(6): p. 418-428.

24. Lee, W.M., et al., *Efficacy of Nutritional Education in Non-Alcoholic Fatty Liver Disease Patients with Hyperlipidemia: a Randomized Controlled Trial.* Gut and liver, 2021. **15**: p. 186.

25. Lustigman, H., O. Tirosh, and A. Shlomai, *Mediterranean Vs. Paleo Diet For The Treatment Of Nonalcoholic Fatty Liver Disease.* Clinical nutrition ESPEN, 2023. **54**: p. 646.

26. Maleki Sedgi, F., M. Mohammad Hosseiniazar, and M. Alizadeh, *The effects of replacing ghee with rapeseed oil on liver steatosis and enzymes, lipid profile, insulin resistance and anthropometric measurements in patients with non-alcoholic fatty liver disease: a randomised controlled clinical trial.* British journal of nutrition, 2024. **131**(12): p. 1985‐1996.

27. Martin, F., et al., *Low hypocaloric mediterranean diet versus standard diet in biopsy proven nafld patients.* Hepatology (Baltimore, Md.), 2021. **74**(SUPPL 1): p. 81A‐82A.

28. Perez-Diaz-del-Campo, N., et al., *Response to a 6-month personalized dietary intervention in patients with metabolic dysfunction-associated steatotic liver disease.* Digestive and liver disease, 2024. **56**: p. S55.

29. Qian, L., et al., *Interventional effect of resistant starch diet on nonalcoholic fatty liver disease, a randomized clinical trial.* Journal of diabetes investigation, 2018. **9**: p. 26‐27.

30. Shidfar, F., et al., *Do symbiotic and Vitamin E supplementation have favorite effects in nonalcoholic fatty liver disease? A randomized, double-blind, placebo-controlled trial.* Journal of gastroenterology and hepatology, 2019. **34**: p. 150.

31. Xiao, Y.X., et al., *Effect of 5:2 Fasting Diet on Liver Fat Content in Patients with Type 2 Diabetic with Nonalcoholic Fatty Liver Disease.* Metabolic syndrome and related disorders, 2022. **20**(8): p. 459-465.

32. Alam, S., et al., *Effect of probiotics supplementation on liver stiffness and steatosis in patients with NAFLD.* Hepatology Forum, 2024. **5**(1): p. 18-24.

33. Deng, Y., et al., *Effects of time-restricted eating on intrahepatic fat and metabolic health among patients with nonalcoholic fatty liver disease.* Obesity, 2024. **32**(3): p. 494-505.

34. Rinaldi, R., et al., *Gender Differences in Liver Steatosis and Fibrosis in Overweight and Obese Patients with Metabolic Dysfunction-Associated Steatotic Liver Disease before and after 8 Weeks of Very Low-Calorie Ketogenic Diet.* Nutrients, 2024. **16**(10).

35. Alferink, L.J.M., et al., *Adherence to a plant-based, high-fibre dietary pattern is related to regression of non-alcoholic fatty liver disease in an elderly population.* European Journal of Epidemiology, 2020. **35**(11): p. 1069-1085.

36. Badran, H., et al., *Impact of intermittent fasting on laboratory, radiological, and anthropometric parameters in NAFLD patients.* Clin Exp Hepatol, 2022. **8**(2): p. 118-124.

37. Belopolsky, Y., et al., *Ketogenic, hypocaloric diet improves nonalcoholic steatohepatitis.* Journal of Translational Internal Medicine, 2020. **8**(1): p. 26-31.

38. Biolato, M., et al., *Intestinal permeability after Mediterranean diet and low-fat diet in non-alcoholic fatty liver disease.* World journal of gastroenterology, 2019. **25**(4): p. 509‐520.

39. De Nucci, S., et al., *Effects of an Eight Week Very Low-Calorie Ketogenic Diet (VLCKD) on White Blood Cell and Platelet Counts in Relation to Metabolic Dysfunction-Associated Steatotic Liver Disease (MASLD) in Subjects with Overweight and Obesity.* Nutrients, 2023. **15**(20).

40. Ebrahimi, S., et al., *Ramadan fasting improves liver function and total cholesterol in patients with nonalcoholic fatty liver disease.* International Journal for Vitamin and Nutrition Research, 2020. **90**(1-2): p. 95-102.

41. Entezari, M.R., et al., *Mediterranean dietary pattern and non-alcoholic fatty liver diseases: a case-control study.* Journal of Nutritional Science, 2021. **10**.

42. Feehan, J., et al., *Time-Restricted Fasting Improves Liver Steatosis in Non-Alcoholic Fatty Liver Disease-A Single Blinded Crossover Trial.* Nutrients, 2023. **15**(23).

43. George, E.S., et al., *A Mediterranean and low-fat dietary intervention in non-alcoholic fatty liver disease patients: exploring participant experience and perceptions about dietary change.* Journal of human nutrition and dietetics, 2023. **36**(3): p. 592‐602.

44. Hawa, F., et al., *Effective Treatment of Nonalcoholic Fatty Liver Disease Using a Community-Based Weight Management Program.* Cureus Journal of Medical Science, 2021. **13**(7).

45. London, A., et al., *The Impact of Short-term Eucaloric Low-Carbohydrate and High-Carbohydrate Diet on Liver Triacylglycerol Content in Males with Overweight and Obesity; a Randomized Cross-Over Study.* American journal of clinical nutrition, 2024.

46. Mardinoglu, A., et al., *An Integrated Understanding of the Rapid Metabolic Benefits of a Carbohydrate-Restricted Diet on Hepatic Steatosis in Humans.* Cell Metab, 2018. **27**(3): p. 559-571.e5.

47. Mari, A., et al., *The Impact of Ramadan Fasting on Fatty Liver Disease Severity: A Retrospective Case Control Study from Israel.* Isr Med Assoc J, 2021. **23**(2): p. 94-98.

48. Rinaldi, R., et al., *The Effects of Eight Weeks' Very Low-Calorie Ketogenic Diet (VLCKD) on Liver Health in Subjects Affected by Overweight and Obesity.* Nutrients, 2023. **15**(4).

49. Arab, A., et al., *The Effect of Nutrition Education Program on Overweight/Obese Patients with Non-Alcoholic Fatty Liver Disease: a Single-Blind Parallel Randomized Controlled Trial.* Clinical nutrition research, 2019. **8**(3): p. 238‐246.

50. Calabrese, F.M., et al., *A Low Glycemic Index Mediterranean Diet Combined with Aerobic Physical Activity Rearranges the Gut Microbiota Signature in NAFLD Patients.* Nutrients, 2022. **14**(9).

51. Ezpeleta, M., et al., *Alternate-Day Fasting Combined with Exercise: Effect on Sleep in Adults with Obesity and NAFLD.* Nutrients, 2023. **15**(6).

52. Fathi, M., et al., *The effects of zinc supplementation on metabolic profile and oxidative stress in overweight/obese patients with non-alcoholic fatty liver disease: A randomized, double-blind, placebo-controlled trial.* Journal of Trace Elements in Medicine and Biology, 2020. **62**.

53. Fathi, M., et al., *The Effect of Zinc Supplementation on Steatosis Severity and Liver Function Enzymes in Overweight/Obese Patients with Mild to Moderate Non-alcoholic Fatty Liver Following Calorie-Restricted Diet: a Double-Blind, Randomized Placebo-Controlled Trial.* Biological Trace Element Research, 2020. **197**(2): p. 394-404.

54. Franco, I., et al., *Physical Activity and Low Glycemic Index Mediterranean Diet: Main and Modification Effects on NAFLD Score. Results from a Randomized Clinical Trial.* Nutrients, 2020. **13**(1).

55. Freer, C.L., et al., *Delivery of a telehealth supported home exercise program with dietary advice to increase plant-based protein intake in people with non-alcoholic fatty liver disease: a 12-week randomised controlled feasibility trial.* British journal of nutrition, 2024. **131**(10): p. 1709‐1719.

56. Ghaffari, A., et al., *Effects of turmeric and chicory seed supplementation on antioxidant and inflammatory biomarkers in patients with non-alcoholic fatty liver disease (NAFLD).* Advances in integrative medicine, 2018. **5**(3): p. 89‐95.

57. Gheflati, A., E. Adelnia, and A. Nadjarzadeh, *The clinical effects of purslane (Portulaca oleracea) seeds on metabolic profiles in patients with nonalcoholic fatty liver disease: a randomized controlled clinical trial.* Phytotherapy research : PTR, 2019. **33**(5): p. 1501‐1509.

58. Maleki, Z., et al., *Effect of soy milk consumption on glycemic status, blood pressure, fibrinogen and malondialdehyde in patients with non-alcoholic fatty liver disease: a randomized controlled trial.* Complementary therapies in medicine, 2019. **44**: p. 44‐50.

59. Morvaridzadeh, M., et al., *Probiotic Yogurt Fortified with Vitamin D Can Improve Glycemic Status in Non-Alcoholic Fatty Liver Disease Patients: a Randomized Clinical Trial.* Clinical nutrition research, 2021. **10**(1): p. 36‐47.

60. Musazadeh, V., et al., *Omega 3-rich Camelina sativa oil in the context of a weight loss program improves glucose homeostasis, inflammation and oxidative stress in patients with NAFLD: a randomised placebo-controlled clinical trial.* International journal of clinical practice, 2021. **75**(11): p. e14744.

61. Orang, Z., M.A. Mohsenpour, and H. Mozaffari-Khosravi, *Effect of Omega-3 fatty acid supplementation on inflammatory markers and insulin resistance indices in patient with type 2 diabetes and nonalcoholic fatty liver: a randomized double-blind clinical trial.* Obesity medicine, 2020. **19**.

62. Orang, Z., et al., *The effect of omega-3 supplementation on glycemic indices and lipid profile in type 2 diabetic patients with non-alcoholic fatty liver disease: a double-blind, randomized, clinical trial.* Mediterranean journal of nutrition and metabolism, 2019. **12**(2): p. 187‐196.

63. Pintó, X., et al., *A Mediterranean Diet Rich in Extra-Virgin Olive Oil Is Associated with a Reduced Prevalence of Nonalcoholic Fatty Liver Disease in Older Individuals at High Cardiovascular Risk.* Journal of nutrition, 2019. **149**(11): p. 1920‐1929.

64. Reddy, A., et al., *Adherence to a Mediterranean diet may improve serum adiponectin in adults with nonalcoholic fatty liver disease: The MEDINA randomized controlled trial.* Nutrition Research, 2023. **119**: p. 98-108.

65. Vahedi, H., et al., *The effect of sesame oil consumption compared to sunflower oil on lipid profile, blood pressure, and anthropometric indices in women with non-alcoholic fatty liver disease: a randomized double-blind controlled trial.* Trials, 2022. **23**(1).

66. Zare, Z., et al., *The effect of the dietary approaches to stop hypertension diet on total antioxidant capacity, superoxide dismutase, catalase, and body composition in patients with non-alcoholic fatty liver disease: a randomized controlled trial.* Frontiers in nutrition, 2023. **10**: p. 1163516.

67. Daneshi-Maskooni, M., et al., *Green cardamom supplementation improves serum irisin, glucose indices, and lipid profiles in overweight or obese non-alcoholic fatty liver disease patients: a double-blind randomized placebo-controlled clinical trial.* BMC complementary and alternative medicine, 2019. **19**(1): p. 59.

68. Arefhosseini, S., et al., *The effect of hydroxy citric acid supplementation with calorie-restricted diet on metabolic, atherogenic and inflammatory biomarkers in women with non-alcoholic fatty liver disease: a randomized controlled clinical trial.* Food & function, 2022. **13**(9): p. 5124‐5134.

69. Atefi, M., et al., *Sesame Oil Ameliorates Alanine Aminotransferase, Aspartate Aminotransferase, and Fatty Liver Grade in Women with Nonalcoholic Fatty Liver Disease Undergoing Low-Calorie Diet: A Randomized Double-Blind Controlled Trial.* Int J Clin Pract, 2022. **2022**: p. 4982080.

70. Bayram, H.M., R. Iliaz, and F.E. Gunes, *Effects of Cornus mas L. on anthropometric and biochemical parameters among metabolic associated fatty liver disease patients: randomized clinical trial.* Journal of ethnopharmacology, 2024. **318**(Pt B): p. 117068.

71. Charatcharoenwitthaya, P., et al., *Moderate-Intensity Aerobic vs Resistance Exercise and Dietary Modification in Patients With Nonalcoholic Fatty Liver Disease: a Randomized Clinical Trial.* Clinical and translational gastroenterology, 2021. **12**(3): p. e00316.

72. Damavandi, R.D., et al., *Effect of <i>Portulaca Oleracea</i> (purslane) extract on liver enzymes, lipid profile, and glycemic status in nonalcoholic fatty liver disease: A randomized, double-blind clinical trial.* Phytotherapy Research, 2021. **35**(6): p. 3145-3156.

73. de Faria Ghetti, F., et al., *Effects of dietary intervention on gut microbiota and metabolic-nutritional profile of outpatients with non-alcoholic steatohepatitis: a randomized clinical trial.* Journal of Gastrointestinal & Liver Diseases, 2019. **28**(3).

74. Deibert, P., et al., *Comprehensive lifestyle intervention vs soy protein-based meal regimen in non-alcoholic steatohepatitis.* World J Gastroenterol, 2019. **25**(9): p. 1116-1131.

75. Dinu, M., et al., *A Khorasan Wheat-Based Replacement Diet Improves Risk Profile of Patients With Nonalcoholic Fatty Liver Disease (NAFLD): a Randomized Clinical Trial.* Journal of the American College of Nutrition, 2018. **37**(6): p. 508‐514.

76. Farzin, L., et al., *No beneficial effects of resveratrol supplementation on atherogenic risk factors in patients with nonalcoholic fatty liver disease.* International Journal for Vitamin and Nutrition Research, 2020. **90**(3-4): p. 279-289.

77. He, Z., et al., *Effects of Oral Vitamin C Supplementation on Liver Health and Associated Parameters in Patients With Non-Alcoholic Fatty Liver Disease: A Randomized Clinical Trial.* Frontiers in nutrition, 2021. **8**: p. 745609.

78. Jin, Y.F., et al., *Silymarin decreases liver stiffness associated with gut microbiota in patients with metabolic dysfunction-associated steatotic liver disease: a randomized, double-blind, placebo-controlled trial.* Lipids in Health and Disease, 2024. **23**(1).

79. Kedarisetty, C.K., et al., *Efficacy of combining pentoxiphylline and vitamin E versus vitamin E alone in non-alcoholic steatohepatitis- A randomized pilot study.* Indian journal of gastroenterology, 2021. **40**(1): p. 41‐49.

80. Masnadi Shirazi, K., et al., *Effect of cranberry supplementation on liver enzymes and cardiometabolic risk factors in patients with NAFLD: a randomized clinical trial.* BMC Complement Med Ther, 2021. **21**(1): p. 283.

81. Milkarizi, N., et al., *Effects of <i>Portulaca oleracea</i> (purslane) on liver function tests, metabolic profile, oxidative stress and inflammatory biomarkers in patients with non-alcoholic fatty liver disease: a randomized, double-blind clinical trial.* Frontiers in Nutrition, 2024. **11**.

82. Ni, Y.Q., et al., *Resistant starch decreases intrahepatic triglycerides in patients with NAFLD via gut microbiome alterations.* Cell Metabolism, 2023. **35**(9): p. 1530-+.

83. Rezaei, S., et al., *Olive oil lessened fatty liver severity independent of cardiometabolic correction in patients with non-alcoholic fatty liver disease: A randomized clinical trial.* Nutrition, 2019. **57**: p. 154-161.

84. Rezaei, S.M.A., et al., *The effects of zinc supplementation on the metabolic factors in patients with non-alcoholic fatty liver disease: a randomized, double-blinded, placebo-controlled clinical trial.* BMC nutrition, 2023. **9**(1): p. 138.

85. Sun, P., et al., *Effect of a High Protein, Low Glycemic Index Dietary Intervention on Metabolic Dysfunction-Associated Fatty Liver Disease: A Randomized Controlled Trial.* Frontiers in Nutrition, 2022. **9**.

86. Daneshi-Maskooni, M., et al., *Green cardamom increases Sirtuin-1 and reduces inflammation in overweight or obese patients with non-alcoholic fatty liver disease: a double-blind randomized placebo-controlled clinical trial.* Nutrition & metabolism, 2018. **15**(1): p. 63.

87. Brandhorst, S., et al., *Fasting-mimicking diet causes hepatic and blood markers changes indicating reduced biological age and disease risk.* Nat Commun, 2024. **15**(1): p. 1309.

88. Cicero, A.F.G., et al., *Effects of phytosomal curcumin on anthropometric parameters, insulin resistance, cortisolemia and non-alcoholic fatty liver disease indices: a double-blind, placebo-controlled clinical trial.* European journal of nutrition, 2020. **59**(2): p. 477‐483.

89. Crabtree, C.D., et al., *Comparison of Ketogenic Diets with and without Ketone Salts versus a Low-Fat Diet: liver Fat Responses in Overweight Adults.* Nutrients, 2021. **13**(3).

90. Cunha, G.M., et al., *Efficacy of a 2-Month Very Low-Calorie Ketogenic Diet (VLCKD) Compared to a Standard Low-Calorie Diet in Reducing Visceral and Liver Fat Accumulation in Patients With Obesity.* Frontiers in Endocrinology, 2020. **11**.

91. Curci, R., et al., *The Effect of Low Glycemic Index Mediterranean Diet and Combined Exercise Program on Metabolic-Associated Fatty Liver Disease: A Joint Modeling Approach.* Journal of Clinical Medicine, 2022. **11**(15).

92. Della Pepa, G., et al., *Effects of a multifactorial ecosustainable isocaloric diet on liver fat in patients with type 2 diabetes: randomized clinical trial.* Bmj Open Diabetes Research & Care, 2020. **8**(1).

93. De Nucci, S., et al., *The Replacement of Only One Portion of Starchy Carbohydrates with Green Leafy Vegetables Regresses Mid and Advanced Stages of NAFLD: Results from a Prospective Pilot Study.* Nutrients, 2023. **15**(10).

94. Dong, T.S., et al., *The Intestinal Microbiome Predicts Weight Loss on a Calorie-Restricted Diet and Is Associated With Improved Hepatic Steatosis.* Frontiers in Nutrition, 2021. **8**.

95. Gepner, Y., et al., *The beneficial effects of Mediterranean diet over low-fat diet may be mediated by decreasing hepatic fat content.* Journal of Hepatology, 2019. **71**(2): p. 379-388.

96. Goss, A.M., et al., *Effects of a carbohydrate-restricted diet on hepatic lipid content in adolescents with nonalcoholic fatty liver disease.* Diabetes, 2019. **68**.

97. Green, C.J., et al., *Hepatic de novo lipogenesis is suppressed and fat oxidation is increased by omega-3 fatty acids at the expense of glucose metabolism.* BMJ open diabetes research & care, 2020. **8**(1).

98. Kahleova, H., et al., *Effect of a Low-Fat Vegan Diet on Body Weight, Insulin Sensitivity, Postprandial Metabolism, and Intramyocellular and Hepatocellular Lipid Levels in Overweight Adults: A Randomized Clinical Trial.* JAMA Netw Open, 2020. **3**(11): p. e2025454.

99. Luukkonen, P.K., et al., *Effect of a ketogenic diet on hepatic steatosis and hepatic mitochondrial metabolism in nonalcoholic fatty liver disease.* Proceedings of the National Academy of Sciences of the United States of America, 2020. **117**(13): p. 7347-7354.

100. Ministrini, S., et al., *Lysosomal Acid Lipase as a Molecular Target of the Very Low Carbohydrate Ketogenic Diet in Morbidly Obese Patients: The Potential Effects on Liver Steatosis and Cardiovascular Risk Factors.* J Clin Med, 2019. **8**(5).

101. Nagy, E.N., et al., *The Effect of Laser Therapy Along With Mediterranean Diet Versus Mediterranean Diet Only on Older Adults With Non-alcoholic Fatty Liver Disease: A Randomized Clinical Trial.* Journal of Lasers in Medical Sciences, 2021. **12**.

102. Parker, A. and Y. Kim, *The Effect of Low Glycemic Index and Glycemic Load Diets on Hepatic Fat Mass, Insulin Resistance, and Blood Lipid Panels in Individuals with Nonalcoholic Fatty Liver Disease.* Metab Syndr Relat Disord, 2019. **17**(8): p. 389-396.

103. Salas-Salvadó, J., et al., *Effect of a Lifestyle Intervention Program With Energy-Restricted Mediterranean Diet and Exercise on Weight Loss and Cardiovascular Risk Factors: One-Year Results of the PREDIMED-Plus Trial.* Diabetes Care, 2019. **42**(5): p. 777-788.

104. Sangouni, A.A., M. Hosseinzadeh, and K. Parastouei, *The effect of dietary approaches to stop hypertension (DASH) diet on fatty liver and cardiovascular risk factors in subjects with metabolic syndrome: a randomized controlled trial.* BMC Endocr Disord, 2024. **24**(1): p. 126.

105. Schutte, S., et al., *A 12-wk whole-grain wheat intervention protects against hepatic fat: the Graandioos study, a randomized trial in overweight subjects.* American journal of clinical nutrition, 2018. **108**(6): p. 1264‐1274.

106. Shidfar, F., et al., *The Effects of Extra Virgin Olive Oil on Alanine Aminotransferase, Aspartate Aminotransferase, and Ultrasonographic Indices of Hepatic Steatosis in Nonalcoholic Fatty Liver Disease Patients Undergoing Low Calorie Diet.* Canadian Journal of Gastroenterology and Hepatology, 2018. **2018**.

107. Sila, A., et al., *Higher-Level Steatosis Is Associated with a Greater Decrease in Metabolic Dysfunction-Associated Steatoic Liver Disease after Eight Weeks of a Very Low-Calorie Ketogenic Diet (VLCKD) in Subjects Affected by Overweight and Obesity.* Nutrients, 2024. **16**(6).

108. Thomsen, M.N., et al., *Dietary carbohydrate restriction augments weight loss-induced improvements in glycaemic control and liver fat in individuals with type 2 diabetes: a randomised controlled trial.* Diabetologia, 2022. **65**(3): p. 506-517.

109. Tsaban, G., et al., *The effect of green Mediterranean diet on cardiometabolic risk; a randomised controlled trial.* Heart, 2021. **107**(13): p. 1054-1061.

110. Wernicke, C., et al., *Effect of unsaturated fat and protein intake on liver fat in people at risk of unhealthy aging: 1-year results of a randomized controlled trial.* American Journal of Clinical Nutrition, 2023. **117**(4): p. 785-793.

111. Willmann, C., et al., *Potential effects of reduced red meat compared with increased fiber intake on glucose metabolism and liver fat content: a randomized and controlled dietary intervention study.* American journal of clinical nutrition, 2019. **109**(2): p. 288‐296.

112. Xu, C.C., et al., *High-protein diet more effectively reduces hepatic fat than low-protein diet despite lower autophagy and FGF21 levels.* Liver international, 2020. **40**(12): p. 2982-2997.

113. Yahay, M., et al., *The effects of canola and olive oils consumption compared to sunflower oil, on lipid profile and hepatic steatosis in women with polycystic ovarian syndrome: a randomized controlled trial.* Lipids in health and disease, 2021. **20**(1): p. 7.


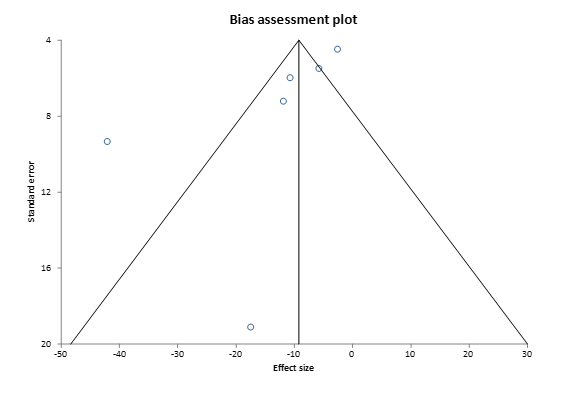


Fig. 8 Funnel Plot assessing potential publication bias regarding the effect of fasting interventions on serum ALT in individuals with MASLD. The vertical line represents the pooled effect size, and the diagonal lines indicate the 95% confidence limits.

**
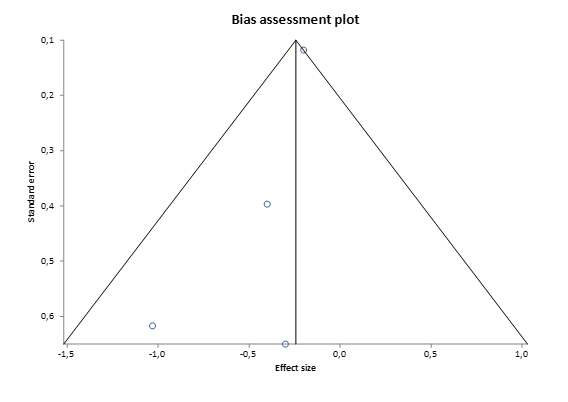
**

# Fig. 9 Funnel plot assessing potential publication bias regarding the effect of fasting interventions on liver stiffness in individuals with MASLD. The vertical line represents the pooled effect size, and the diagonal lines indicate the 95% confidence limits.


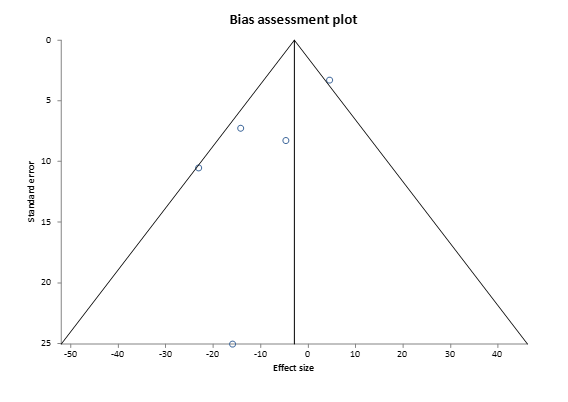


# Fig. 10 Funnel Plot assessing potential publication bias regarding the effect of a LCHF/ketogenic diet on serum ALT in individuals with MASLD. The vertical line represents the pooled effect size, and the diagonal lines indicate the 95% confidence limits.

**
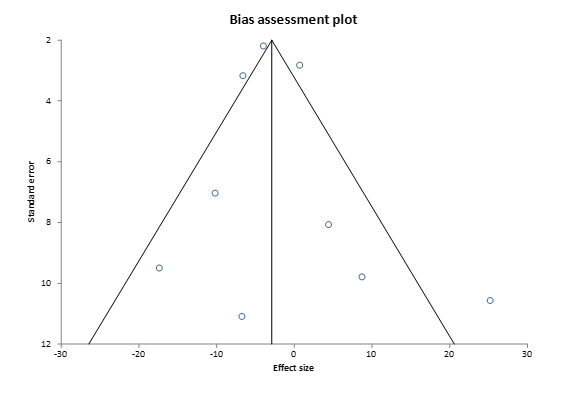
**

# Fig. 11 Funnel Plot assessing potential publication bias regarding the effect of the Mediterranean diet on serum ALT levels in individuals with MASLD. The vertical line represents the pooled effect size, and the diagonal lines indicate the 95% confidence limits.


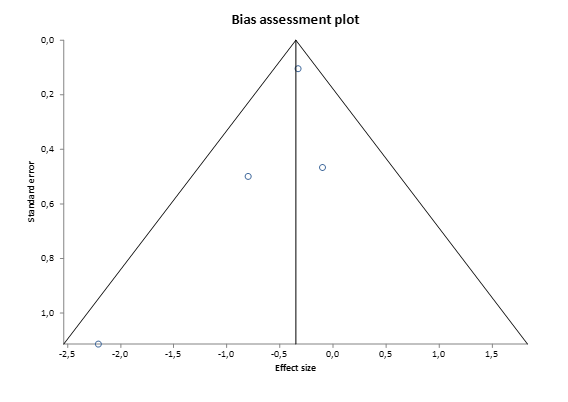


# Fig. 12 Funnel Plot assessing potential publication bias regarding the effect of the Mediterranean diet on liver stiffness in individuals with MASLD. The vertical line represents the pooled effect size, and the diagonal lines indicate the 95% confidence limits.


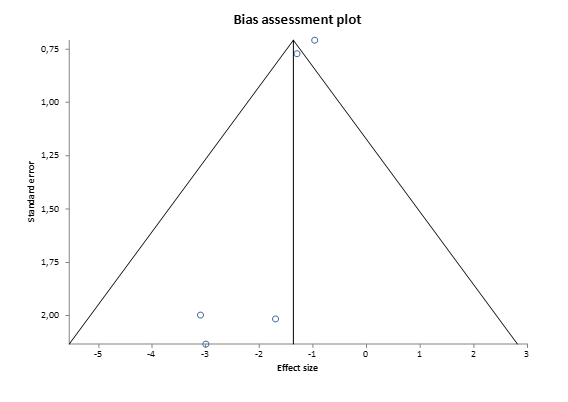


# Fig. 13 Funnel Plot assessing potential publication bias regarding the effect of the Mediterranean diet on MRI-PDFF in individuals with MASLD. The vertical line represents the pooled effect size, and the diagonal lines indicate the 95% confidence limits.

**
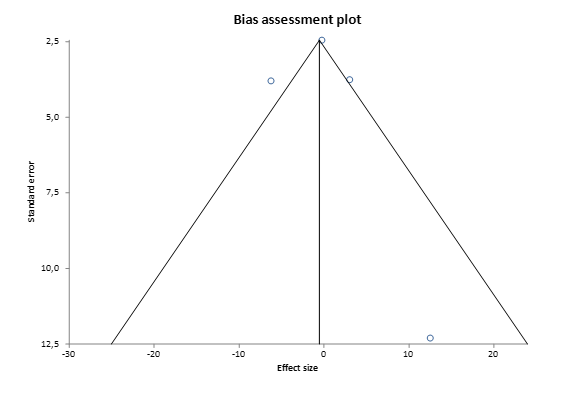
**

# Fig. 14 Funnel Plot assessing potential publication bias regarding the effect of the supplementation of omega-3 fatty acids on serum ALT levels in individuals with MASLD. The vertical line represents the pooled effect size, and the diagonal lines indicate the 95% confidence limits.

**
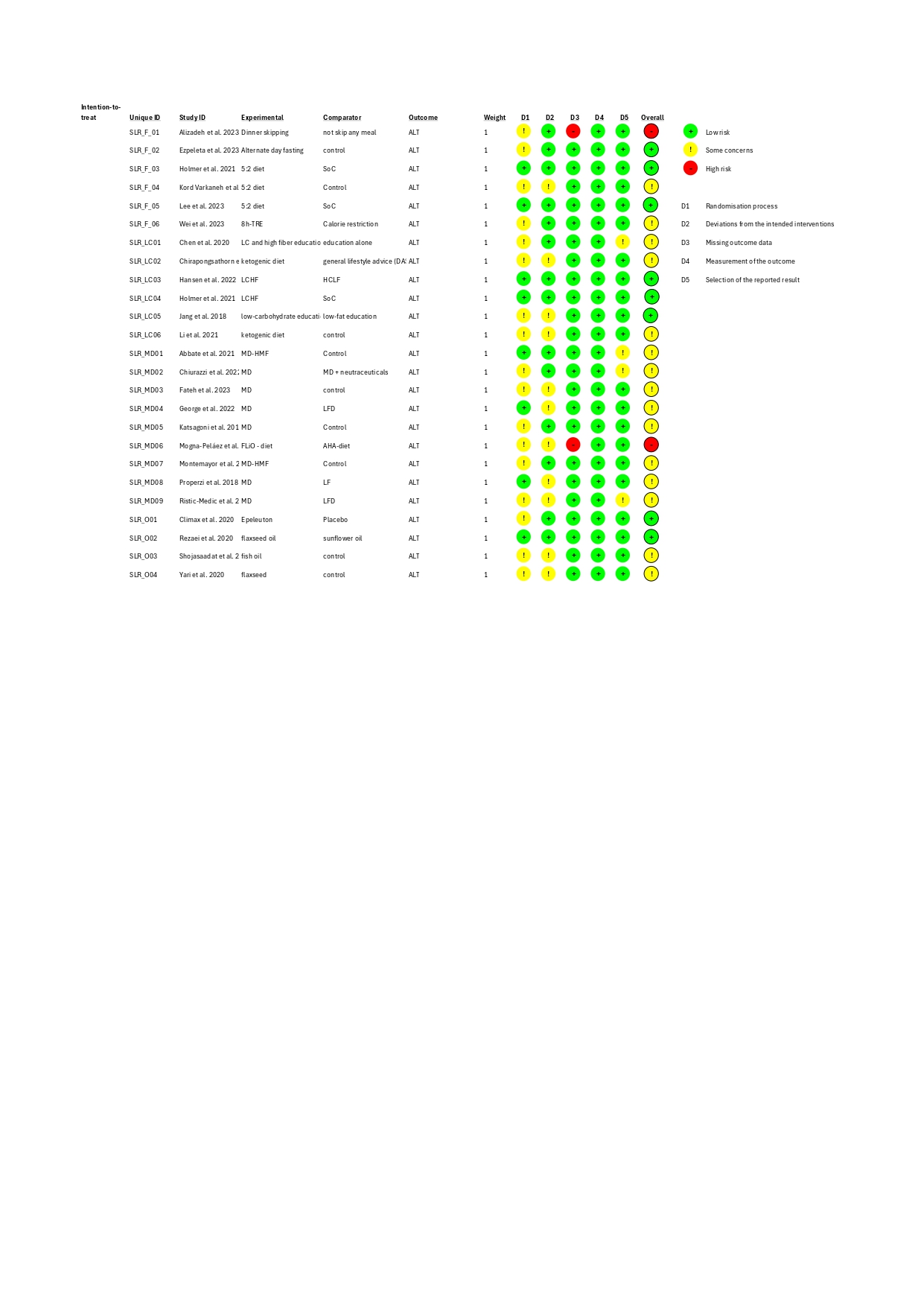
**

# Fig. 15 Quality assessment of the included randomized controlled trials.

# Table 6 GRADE assessment

| **Certainty assessment** | | | | | | | **№ of patients** | | **Effect** | | **Certainty** |
| --- | --- | --- | --- | --- | --- | --- | --- | --- | --- | --- | --- |
| **№ of studies** | **Study design** | **Risk of bias** | **Inconsistency** | **Indirectness** | **Imprecision** | **Other considerations** | **fasting** | **control** | **Relative (95% CI)** | **Absolute (95% CI)** |  |
| **Fasting: Reduction of alanine aminotransferase** | | | | | | | | | | | |
| 6 | randomised trials | serious^1,a^ | not serious | not serious | not serious | none | 160 | 154 | - | MD **12.47 IU/L lower** (22.03 lower to 2.92 lower) | ⨁⨁⨁◯ Moderate^1,a^ |
| **Fasting: Reduction of liver stiffness** | | | | | | | | | | | |
| 4 | randomised trials | not serious | not serious | not serious | not serious | none | 109 | 108 | - | MD **0.24 kPa lower** (0.46 lower to 0.03 lower) | ⨁⨁⨁⨁ High |
| **LCHF/ketogenic diet: Reduction of alanine aminotransferase** | | | | | | | | | | | |
| 6 | randomised trials | not serious | not serious | not serious | not serious | none | 186 | 138 | - | MD **6.87 IU/L lower** (15.93 lower to 2.21 higher) | ⨁⨁⨁⨁ High |
| **Mediterranean diet: Reduction of alanine aminotransferase** | | | | | | | | | | | |
| 9 | randomised trials | serious^2,a^ | not serious | not serious | not serious | none | 291 | 295 | - | MD **2.93 IU/L lower** (5.68 lower to 0.19 lower) | ⨁⨁⨁◯ Moderate^2,a^ |
| **Mediterranean diet: Reduction of liver stiffness** | | | | | | | | | | | |
| 4 | randomised trials | serious^2,a^ | not serious | not serious | not serious | none | 155 | 155 | - | MD **0.35 kPa lower** (0.55 lower to 0.16 lower) | ⨁⨁⨁◯ Moderate^2,a^ |
| **Mediterranean diet: Reduction of MRI-PDFF** | | | | | | | | | | | |
| 5 | randomised trials | serious^2,a^ | not serious | not serious | not serious | none | 178 | 180 | - | MD **1.34 % lower** (2.23 lower to 0.4 lower) | ⨁⨁⨁◯ Moderate^2,a^ |
| **Omega-3 fatty acids supplementation: Reduction of alanine aminotransferase** | | | | | | | | | | | |
| 4 | randomised trials | not serious | not serious | not serious | not serious | none | 121 | 117 | - | MD **0.55 IU/L lower** (4.06 lower to 2.96 higher) | ⨁⨁⨁⨁ High |

**CI:** confidence interval; **MD:** mean difference

#### Explanations

a. one study is judged high risk of bias due to missing outcome data

#### References

1. Alizadeh,et al. The effects of meal patterns on liver steatosis, fibrosis and biochemical factors in patients with NAFLD: a RCT.2024.

2. Mogna-Peláez,et al. Inflammatory markers as diagnostic and precision nutrition tools for metabolic dysfunction-associated steatotic liver disease: Results from the Fatty liver in obesity trial.2024.
